# Evolution of switchable aposematism: insights from individual-based simulations

**DOI:** 10.7717/peerj.8915

**Published:** 2020-04-10

**Authors:** Woncheol Song, Sang-im Lee, Piotr G. Jablonski

**Affiliations:** 1 Laboratory of Behavioral Ecology and Evolution, School of Biological Sciences, Seoul National University, Seoul, South Korea; 2 School of Undergraduate Studies, Daegu-Gyeongbuk Institute of Science and Technology, Daegu, South Korea; 3 Museum and Institute of Zoology, Polish Academy of Sciences, Warsaw, Poland

**Keywords:** Aposematism, Startle, Deimatism, Simulation, Model, Evolution, Switchable, Post-attack, Pre-attack

## Abstract

Some defended prey animals can switch on their normally hidden aposematic signals. This switching may occur in reaction to predators’ approach (pre-attack signals) or attack (post-attack signals). Switchable aposematism has been relatively poorly studied, but we can expect that it might bring a variety of benefits to an aposmetic organism. First, the switching could startle the predators (deimatism). Second, it could facilitate aversive learning. Third, it could minimize exposure or energetic expense, as the signal can be switched off. These potential benefits might offset costs of developing, maintaining and utilizing the switchable traits. Here we focused on the third benefit of switchability, the cost-saving aspect, and developed an individual-based computer simulation of predators and prey. In 88,128 model runs, we observed evolution of permanent, pre-attack, or post-attack aposematic signals of varying strength. We found that, in general, the pre-attack switchable aposematism may require moderate predator learning speed, high basal detectability, and moderate to high signal cost. On the other hand, the post-attack signals may arise under slow predator learning, low basal detectability and high signal cost. When predator population turnover is fast, it may lead to evolution of post-attack aposematic signals that are not conforming to the above tendency. We also suggest that a high switching cost may exert different selection pressure on the pre-attack than the post-attack switchable strategies. To our knowledge, these are the first theoretical attempts to systematically explore the evolution of switchable aposematism relative to permanent aposematism in defended prey. Our simulation model is capable of addressing additional questions beyond the scope of this article, and we open the simulation software, program manual and source code for free public use.

## Introduction

### Switchable aposematism

Some prey animals possess defensive measures that make them unprofitable to predators. Such defended prey often signals their unpalatability via bright colors, alarming sounds, or other conspicuous components. These signals can affect predator’s decision to ingest the prey because the predator learns association between the signal and the prey unprofitability. This form of anti-predatory strategy is called aposematism. Evolutionary biologists have explored various dimensions of aposematism, but its behavioral aspect had not received adequate attention until recent years. While some aposematic signals such as permanent coloration are fixed and operate continuously, other signals can be behaviorally controlled, by sound generation (Dowdy & Conner, 2016), wing movement (Kang et al., 2016), bioluminescence (De Cock & Matthysen, 1999), physiological color change (Umbers et al., 2014), or postural change (Lariviere & Messier, 1996). We call this form “switchable aposematism”.

Historically, switchable aposematism has been described with various adjectives such as “facultative” (Sivinski, 1981; Grober, 1988), “post-attack” (Umbers & Mappes, 2015; Kang et al., 2016), “early-acting” (Broom, Higginson & Ruxton, 2010), “deimatic” (Umbers, Lehtonen & Mappes, 2015; Umbers & Mappes, 2015), or “switchable” (Umbers et al., 2017), implying various functional or temporal characteristics found in each study species. This had led to disputes regarding the use of “deimatic” as either descriptor of function or that of a form (Umbers, Lehtonen & Mappes, 2015; Umbers & Mappes, 2015; Skelhorn, Holmes & Rowe, 2016; Umbers et al., 2017). In this article, we chose to use a term that indisputably concerns the form of the display behavior: “switchable”.

### The renewed interest in switchable aposematism

In contrast to the well-developed knowledge on switchable displays in undefended (profitable) prey in the context of “startling” or “deimatic” function (sudden display surprising the predator away: Edmunds (1972), Schlenoff (1985), Grandcolas & Desutter-Grandcolas (1998), Vallin et al. (2005), Bura et al. (2011)), switchable aposematic signals of defended prey have been largely ignored. Notable exceptions are early studies on bioluminescence in defended prey (Sivinski, 1981; Grober, 1988) and short descriptions of switchable conspicuous signals in some defended animals (Robinson, 1969).

Recently, however, researchers experimentally explored the switchable visual displays of some chemically defended prey (Kang, Lee & Jablonski, 2011; Umbers & Mappes, 2015; Kang et al., 2016) rising new questions and “rejuvenating” old ones in this field (Umbers et al., 2017).

### Costs and benefits of switchable aposematism

Physiological and/or anatomical mechanisms that allow signal switching might impose additional costs to the prey animals. Some of these costs might involve development and maintenance of such mechanism (“fixed” cost), while others might be paid every time the switching behavior occurs (“per-use” cost, terminologies adopted from Broom, Higginson & Ruxton (2010)).

On the other hand, a switchable signal can be beneficial in several ways. First, regardless of any aposematic function, a sudden switch may simply surprise the predator away (the “startling” effect; Ruxton et al., 2018). The sudden switching might also accelerate the predator’s learning about the aposematic prey (Kang et al., 2016). Finally, it might cut down unnecessary signal exposure because an animal can present the expensive signal only when needed (Grober, 1988; De Cock & Matthysen, 1999; Umbers et al., 2017).

Among these benefits, the cost-reducing aspect is probably the most cumbersome to handle in empirical studies because the evolutionary cost of a signal is difficult to quantify or manipulate (Srygley, 2004; Bohlin, Tullberg & Merilaita, 2008; Crothers, Gering & Cummings, 2011; Lindstedt et al., 2016). Therefore, we chose to use a modeling approach to focus on the variety of costs of switchable signals.

### Different types of costs related to switchable aposematism

In order to avoid confusion between the different types of costs involved in switchable aposematism, we will use consistent specific expressions from now on. The energetic or material cost paid per every switching will now be called the “switching cost”. The developmental and/or maintenance costs associated to the switching mechanism itself, which is always paid regardless how often the switching-signal is actually fired, will now be collectively called the “switchability maintenance cost”. These two types of costs are expected to hinder the evolution of switchability.

The signal-induced exposure to the predators will now be called the “detectability cost”. All the other costs inherent to the active signal itself, for example, energy consumption for producing sound or potential hindrance in foraging (Brandley, Johnson & Johnsen, 2016) will now be called the “signaling penalty”. These two types of costs are expected to hurdle the evolution of aposematism itself, but switchability may help relieve them. A generic term that encompasses and summarizes all of these negative effects will be the “general cost(s) of signaling”. This general cost is expected to be optimized against the general benefit of signaling. Some of these terminologies will begin to appear in bold texts, indicating that the corresponding concept is directly modeled by a simulation variable.

### Introducing *ApoSim* and its usage in this article

We developed *ApoSim*, an individual-based simulation model to study this subject. In this model, computer-simulated predators and prey interact with each other, and their performances are under selection pressure. *ApoSim* is capable of exploring a very wide parameter space, but for this paper we assumed a special situation; the learning facilitation and startling effects of switchable signals are negligible. Under this assumption, we could explore the cost-reducing aspect of switchable aposematism in a clean, conservative scope. Users of the model software can easily conduct future studies in order to address questions regarding the startle effect (see Supplemental Material).

### Previous knowledge pertaining to the general costs of switchable aposematism

Throughout the development of *ApoSim*, we had to consider a variety of relevant phenomena that have been suggested or reported. The sequential nature of post-attack switching that is dependent on the success of the pre-attack defensive measures (Broom, Higginson & Ruxton, 2010; Ruxton et al., 2018), the baseline detectability of non-signaling prey (De Cock & Matthysen, 1999; Broom, Higginson & Ruxton, 2010), the fixed and per-use costs of switchability (Higginson & Ruxton, 2009; Broom, Higginson & Ruxton, 2010), and sensory and cognitive ability to detect predator’s presence (Broom, Higginson & Ruxton, 2010) have all been under investigation. Notably, the interactions between these mechanisms and the general costs of switchable aposematic signals is not fully understood.

The general cost of switchable signaling is not a single, readily measured value, but a result of highly complex and relatively unexplored interactions. Energy consumption (Srygley, 2004; Lindstedt et al., 2011), predator learning and behavior (Guilford, 1994; Gamberale-Stille & Guilford, 2004; Halpin & Rowe, 2017), the portion of the predators not deterred by the signal (Williams, Brodie & Brodie, 2003; Mappes et al., 2014), the level of crypsis achievable when not signaling (Bohlin, Tullberg & Merilaita, 2008; Willink et al., 2013; Umbers & Mappes, 2015), and many other aspects could all affect the general cost of switchable signaling. Furthermore, some of these conditions can change every time the signal is switched on. Due to this complexity, the study on switchable aposematism is still in its beginning stage. Therefore, we carefully designed the simulation so that it can embrace most of the above ideas in one framework.

### Scope of this study

Based on our simulation, we suggest new hypotheses regarding the evolution of switchable aposematism and test existing hypotheses in a context different from which they were originally formulated. First, we explore if variation in the selective penalty of the aposematic signal is sufficient to explain the observed diversity of switchable aposematism. Second, we determine how evolutionary success of different types of switchable aposematism is affected by increasing costs of switching. Third, we evaluate the existing hypotheses suggesting that moderate-range predator learning capabilities create the most favorable conditions for the evolution of aposematism (Speed, 2001; Puurtinen & Kaitala, 2006). Fourth, we explore some previously proposed hypotheses about the effects of mixing of naïve and experienced predators on the evolution of various aposematic strategies (Endler & Mappes, 2004; Kang, Lee & Jablonski, 2011; Mappes et al., 2014; Kang et al., 2016). Fifth, we determine if and how the basal detectability of the prey affects evolution of aposematism. Finally, we explore several possible evolutionary pathways that can lead to switchable aposematism from non-switching and/or non-aposematic initial population.

Additionally, we provide our model and the source code so that the users can further explore the evolution of complete diversity of aposematic strategies. The model can simulate the interactions between these evolutionary components in a multidimensional evolutionary space. In addition, we provide a description of how to use this software to explore variables other than those used in this article.

## Materials and Methods

### Core ideas and assumptions

#### The prey behavioral strategy

We modeled the prey behavioral strategy as responses to three conditions: “resting,” being “approached” and being “attacked”. Specifically, a strategy is defined by three values of signal intensity (ranging from 0 to 1) each associated with one of the three conditions above. For example, a strategy could be written as the following: ((resting, 0.1), (approached, 0.1), (attacked, 0.7)). Each number represents the signal intensity given in each condition. This strategy is a variant of post-attack switchable signaling, because the sudden increase in signal intensity (from 0.1 to 0.7) occurs once the predator attacks. Meanwhile, a permanently aposematic prey should have the same signal intensity for all three conditions. In the case of a pre-attack switcher, it should remain largely inconspicuous while resting, but switch to higher intensity when “approached.” It should be noted that signal intensity is a one-dimensional value; we assumed that all signal varieties operate on the same common axis, and that the predators automatically generalize their knowledge about one signaling prey to all the others. Therefore, all prey signals are “mimetic” to each other.

While signal intensity could technically take any value from 0 to 1, we further simplified this variable for the sake of easier visualization. We assumed that the prey can only have three discrete levels of signals: “none” (N), “low” (L) and “high” (H) (the exact value of each level can be adjusted by the user). With these discrete levels, one could describe a behavioral strategy without using the numerical values for the signal intensity. For example, the pre-attack switchable aposematism could be written as ((resting, N), (approached, H), (attacked, H)).

We devised a three-letter annotation system as a shorthand for each behavioral strategy. The sequence of the three conditions is easy to remember as they are in the logical order of increasing threat level: resting, approached, and attacked. Therefore, the previous expression could be shortened to (NHH). Likewise, one could easily understand that LLL is a permanent-display strategy with low signal intensity (L). Similarly, NLH is a strategy of an animal that does not signal at rest (N), responds to an approaching predator by switching to a weak pre-attack signal (L), and responds to a predator attack with a stronger signal (H). With this concise style, we could easily express complex ideas such as “competitive advantage of NLL over NLH” or “the change in LLL–NNH balance in the presence of NLLs” without repeating the lengthy explanations every time.

In summary, three signal intensity levels (N, L and H) are allowed in each of the three states (resting, approached and attacked), resulting in 3^3^ = 27 possible behavioral strategies. The user could configure whether a specific strategy can exist or not in the model.

#### The predator attack event

Given the basic structure of the prey behavioral strategies, it was clear that the predator should have at least two attack opportunities. The post-attack switchable aposematic signal should be fired after an attack event, and it should affect the prey survival by influencing the chance of the subsequent attack(s).

Therefore, we assumed that there are initial attack and subsequent attack events. An initial attack event occurs after the prey’s pre-attack behavior and before the post-attack behavior; a subsequent attack event occurs after the post-attack behavior. These two attack events act as junctions that naturally divide the whole interaction into three time steps: pre-initial-attack period (step 1), inter-attack period (step 2), and the post-final-attack period (step 3).

#### The scope of the simulated evolution

The model simulates competition for higher survival among different prey behavioral strategies. In contrast, properties of the predator population are directly specified by the user. Hence, rather than simulating the predator-prey coevolution, the model focuses on the evolution of prey population given a certain fixed set of predator properties. With this approach, we could explore the direct effect of specific characteristics of the predatory guild.

#### Overview of the model structure

We built the model with NetLogo 5.3.1, an agent-based modeling framework developed at Northwestern University (Wilensky, 1999). The simulation code and graphical interface are published with the article. Note that the graphical interface was used only for testing, prototyping and pedagogical use; the exploration of the parameter space was programmatically executed by *BehaviorSpace*, a parameter-search tool bundled with NetLogo 5.3.1.

Our model, *ApoSim*, is capable of modeling startle and learning facilitation effects of switchable signals as well as a number of other ecological variables. However, here we describe only the mechanics relevant to our study questions. For the full description of the model variables, please refer to the Supplemental Materials and the source code.

**Figure 1:**
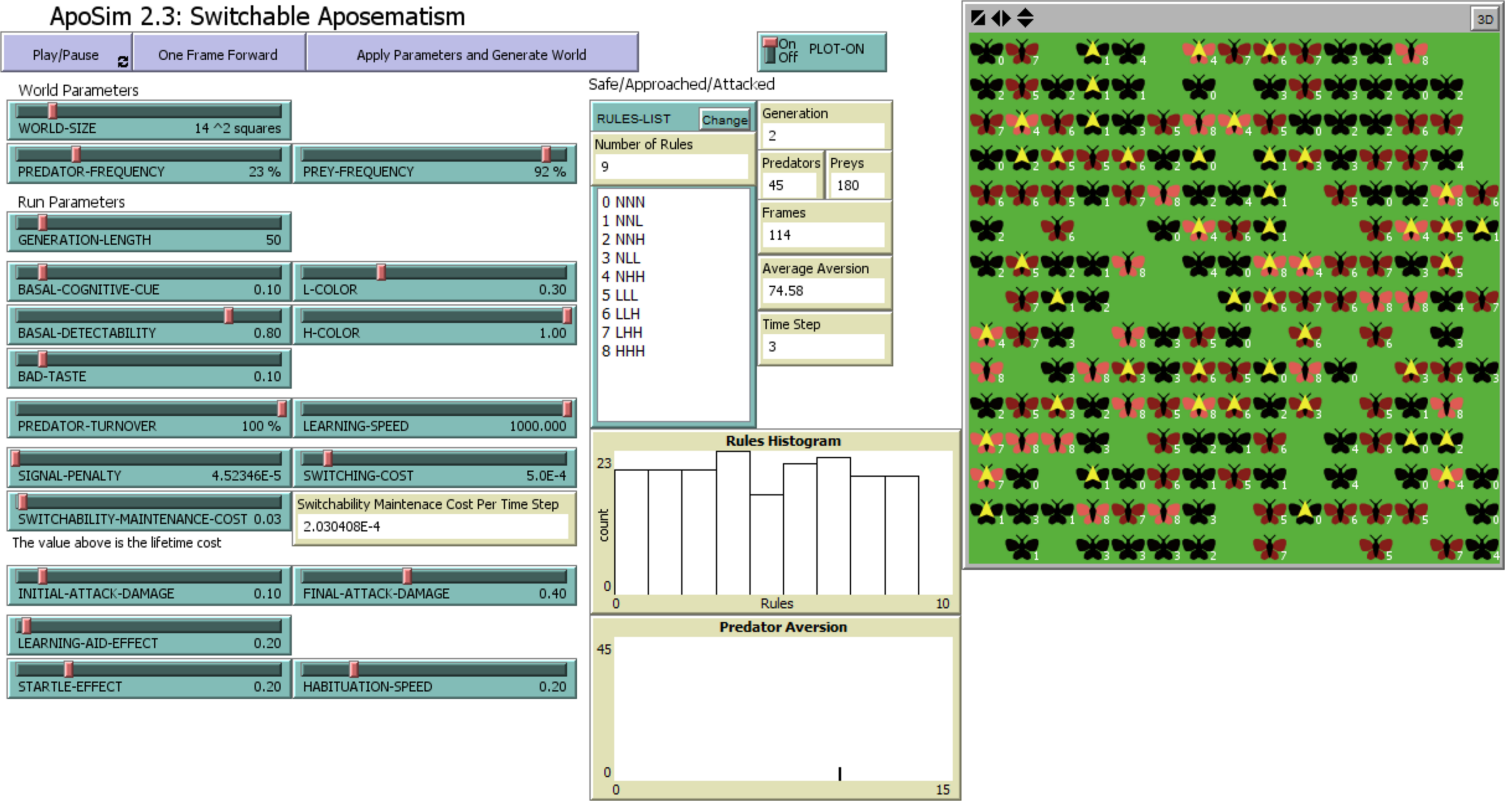
The *ApoSim* model interface built with NetLogo. Within the the model interface panels, the user can design the list of rules and adjust model variables using sliders and input windows. The model status at each moment of simulation is reported in histogram plots and summary statistics. The continuously updated animated view of the two-dimensional world is also shown.

The modeled world consists of a single species of 180 defended prey animals and a single species of 45 predators (Fig. 1). Each individual prey occupies one grid square of the 2-dimensional world (14 × 14 grids). The prey distribution is globally random, and the model does not aim to simulate the kin-selection effect of gregariousness. Initially the prey population is a uniform mix of all possible behavioral strategies.

The basic time unit of the simulation is the interaction frame (Table 1). A total of 50 interaction frames forms one prey generation, after which the current prey individuals reproduce and die out. Each interaction frame includes three time steps. In each time step, every prey and predator individual follows specific set of steps coded in program’s procedures. The names of the procedures, as they appear in the source code, are given in Table 1 and are detailed in the subsequent sections.

**Table 1:** The modeled predator–prey interaction timeline.

Prey generation	Interaction frame	Time step	Program procedures
Generation 1	Frame 1	Step 1	Predators-move Prey-react Prey-pay-cost Predators-search Predators-observe Predators-analyze Predators-decide
		Step 2	Predators-initial-act Predators-feel Prey-react Prey-pay-cost Predators-observe Predators-learn Predators-analyze Predators-decide
		Step 3	Predators-final-act Predators-feel Prey-react Prey-pay-cost Predators-observe Predators-learn
	Frame 2 … Frame 50	(Repeat the three time steps for each interaction frame)	
	Reproduction stage		Prey-selectively-die Prey-lay All-prey-die prevent-extinction Predators-turnover Prey-emerge
Generation 2 … Generation 500	(Repeat the 50 interaction frames and the reproduction stage for each generation)		

During each interaction frame, a predator could encounter and interact with an individual prey throughout the three time steps. During the interaction, the predator can detect the prey’s repulsive taste, which reduces the predator motivation. The exposure to repulsive taste could lead to accumulation of the predator’s aversive memory value from 0 to unlimited level. After enough interactions, the heightened level of aversive memory could cause the predator to refuse to attack this prey species. Also, the interaction could lower the survival chance of the prey, which begins as 1 and then subsequently decreases by multiplying by decimal modifiers. Actions of the predator and the prey determines the levels of the modifiers. Depending on how the interaction unfolds, the predator might fail to detect the prey or decide to abandon it. In such a case, the interaction is prematurely terminated, and the predator would idle for the rest of the interaction frame.

After 50 interaction frames (one prey generation) all prey enter the reproduction stage. First, the prey individuals die out according to the final values of survival chance. All the survivors have equal chance of reproduction. Reproduction is simulated by repeating the procedure of randomly choosing one of the survivors and generating one offspring of the same behavioral strategy until the carrying capacity (180 individuals) of the next generation is reached. After the entire reproduction stage is over, the survivors of the current generation dies immediately (non-overlapping generations). There is no simulation of offspring growth stages. There is no sexual reproduction, and each offspring exhibits the parental phenotype identically.

On the other hand, the predators do not have any fixed lifespan, and a preset number of fresh naïve predators randomly replace the old individuals according to the user-defined predator turnover rate at the end of each prey generation. We assumed that predators acquire the avoidance entirely through associative learning, not instinct. The predator learning speed is adjustable by the user. The predators are genetically homogeneous and not subject to natural selection.

### Detailed simulation structure

#### Interaction frame: time step 1

“Predators-move” procedure—At the beginning of each interaction frame, every predator moves to a random grid square which contains a prey. In our setup, the number of the predators is smaller than that of prey; this procedure leaves the majority of the prey without predator encounter. Every prey is still in the “resting” stage.

“Prey-react” procedure—Each prey detects the predator presence or absence in the grid square it is occupying, and it then switches or maintains its aposematic signal. After detecting the predator absence or presence, the prey then stays still or switches its signal intensity depending on its behavioral strategy. More specifically, each prey continues to be in “resting” state if no predator is in its grid square, and if one is present, then the prey attains the “approached” state instead. As detailed in the first section of “Methods”, the prey signal intensity is one of the three levels: N, L, or H. Each level corresponds to a number ranging from 0 (minimum possible signal) to 1 (maximum possible signal), and the exact values could be set by the user.

In this study, we assumed that the prey had perfect sensory and cognitive ability to accurately determine the predator presence. We do recognize that ambush predators are major exceptions to this assumption, and therefore we do not intend to explain their ecology with our model.

“Prey-pay-cost” procedure—Regardless whether it is encountering a predator or not, each prey has to pay the energetic and ecological price of its current behavior and appearance. Each prey is continuing its “resting” or “approached” state, and the cost-paying procedure runs like the following.

First, if the prey has switched the signal to a different level, such behavior might require extra energy and reduce its chance of survival. The user-adjusted variable switching cost (range 0–1) represents this effect, and the prey survival chance (range 0–1; initially at 1) decreases accordingly (Eq. (1)). Note that we are using Roman numerals to mark the intermediate values used in sequential calculations (an example is “survival chance I” below). Also, we decided to use full-word names of the variables instead of single letter symbols because we think this will facilitate better and easier understanding of the whole model by the intended readership—biologists who study aposematism.

(1) $${\rm survival\ chance\ I = current\ survival\ chance*(1-switching\ cost)}$$

Second, an animal with a switching mechanism needs to maintain it energetically and cope with the developmental complexity regardless whether the actual switching behavior has fired or not. In our model, a user-adjusted variable switchability maintenance cost (range 0–1) simulated this effect (Eq. (2)).

(2) $${\rm survival\ chance\ II = survival\ chance\ I * (1 - switchability\ maintenance\ cost)}$$

Finally, an active state of the signal itself, be it switchable or permanent, might impose a variety of burden of prey survival that are not explicitly modeled in our software. For instance, high signal intensity could attract parasites that are not deterred by the unpalatability. If the prey’s mode of signaling involved sound generation or odor production, there would be extra energy consumption. If the prey uses a physical device that can be shown, moved, or inflated, the prey animal’s mobility would suffer in addition to energy costs. A myriad of such effects are summarized in our model as signaling penalty, a user-adjustable value from 0 to 1. This is weighted by signal intensity (Eq. (3)).

(3) $${\rm new\ survival\ chance = survival\ chance\ II * (1 - signaling\ penalty * signal\ intensity)}$$

In principle, there would be a production and maintenance cost for all signals regardless switchability; some developmental burdens such as pigment production could be shared by permanent and switchable signals. We decided to exclude this variable from the model, as its effect is largely trivial and predictable—the costlier the signal maintenance, the lower the prevalence of signaling phenotypes.

“Predators-search” procedure—Each predator investigates the grid square and detects the prey presence with a certain probability. This discovery chance (Eq. 4) is a function of basal detectability and signal intensity of the prey (which will all be in the “approached” state; only the prey sharing its grid square with a predator can be searched, so no “resting” prey is under consideration here). Basal detectability (range 0–1; user-adjusted variable) is a simplified representation of prey body shape, the habitat properties, the predator cognitive attributes, and anything that can affect the minimum level of discovery chance. Hence, the discovery chance value is larger for higher basal detectability. It is additionally increased if the signal intensity value is larger than 0. The signal intensity effect is weighted by the maximally possible increase of the detectability (1—basal detectability), so the final value is limited between 0 and 1. Unlike many other similar equations in the model, basal detectability and signal intensity have an additive, not a multiplicative, relationship. The rationale for this design is that both basal detectability and signal intensity should be able to contribute to predator discovery even if the other variable is close to zero or zero.

(4) $${\rm discovery\ chance = basal\ detectability + signal\ intensity * (1 - basal\ detectability)}$$

“Predators-observe” procedure—If a predator has detected a prey, then it observes the appearance of the target prey (which is still in its “approached” state). Conditional stimulus (range 0–1) is a value that represented the intensity of all information that can mediate the predator’s associative learning regarding the prey. A high conditional stimulus value means that the prey has characteristics that allow easier memory buildup and recall.

The information available to predator could have been come from either the prey’s signal or its signal-independent inherent traits. Conditional stimulus is a function of prey signal intensity and basal cognitive cue (Eq. (5)). Note that the formula is in the same format as Eq. (4); basal cognitive cue sets the baseline, and signal intensity determines the remaining portion so that the final value cannot exceed 1.

(5) $${\rm conditional\ stimulus = {\rm basal\ cognitive\ cue} + {\rm signal\ intensity} * (1 -\ {\rm basal\ cognitive\ cue})}$$

The prey body shape, the predator cognitive attributes, environmental conditions, and many other factors could govern the minimum level of associative learning and memory retrieval. Basal cognitive cue (range 0–1; user-adjusted variable) represents them. Note that basal detectability and basal cognitive cue are two different variables despite looking similar. Basal detectability affects the chance of discovery (Eq. (4)), while basal cognitive cue is for the learning/recalling stimulus (Eq. (5)). For example, a flying insect might be easily detectable (high basal detectability) but it could be difficult to learn or recall from the appearance, because it looks too similar to many other profitable insects (low basal cognitive cue).

“Predators-analyze” procedure—From the information observed from the prey, the predator attempts to determine its attractiveness based on memory as well as taste (if available). After perceiving a conditional stimulus, each predator assesses prey attractiveness. Prey attractiveness (range 0–1) is a function of learned repulsiveness and instinctive repulsiveness, both with a range from 0 to 1 (Eq. (6)). For a prey to be highly attractive, both learned and instinctive repulsiveness should be close to 0.

(6) $${\rm prey\ attractiveness = (1 - learned\ repulsiveness) * (1 - instinctive\ repulsiveness)}$$

The predator’s impression from the prey is determined by the level of memory, the strength of cues that recall the memory, and the currently felt taste (if available). Learned repulsiveness is determined by the level of the conditional stimulus (Eq. (5)) and the aversive memory. The learned repulsiveness is forced to be within the range of (0, 1), as seen in Eq. (7). Instinctive repulsiveness is solely determined by the unconditional stimulus (range 0–1) the predator is currently feeling (Eq. (8)). Both unconditional stimulus and conditional stimulus are terminologies following the convention in classical conditioning (Sweatt, 2009); they themselves are not “memory”, but an association between them (aversive memory) is. Also, the model software can be configured to include the “startle” effect in the instinctive repulsiveness calculation; see Supplemental Materials.

(7) $${\rm learned\ repulsiveness = minimum} \left\{ {\matrix{ 1 \cr {\rm aversive\ memory*conditional\ stimulus} \cr } } \right.$$

(8) $${\rm instinctive\ repulsiveness = unconditional\ stimulus}$$

The predator’s past memory and the cues that recall it are both necessary for a learned avoidance to take effect. Unlike all the other values that range from 0 to 1, aversive memory could be any non-negative number and had no upper bound. Therefore, sufficiently high aversive memory could bring the learned repulsiveness up to 1 even if the conditional stimulus (Eq. (5)) is very small. On the other hand, a completely naïve predator with no aversive memory, will have no learned repulsiveness regardless of the conditional stimulus. The mechanics of the aversive memory buildup will be detailed in the subsequent sections (Eqs. (12a) and (12b)).

The “taste” information is only accessible after the predator made at least one attack event. The unconditional stimulus represented all modes of unprofitability that can be sensed by the predator only during attack events. In the current time step 1, the predator has not yet tasted the prey (which is still in its “approached” state), so the received unconditional stimulus is 0 (Eq. (8.9)). In the later time steps, there will be taste information available, and the mechanics will be detailed in the subsequent sections.

(8.9) $${\rm unconditional\ stimulus = 0}$$

“Predators-decide” procedure—From the predator’s impression of the target prey (which is still in the “approached” state), the “decision” to attack (or not) is made. Motivation (range 0–1) began from 1 whenever the predator encountered a new prey. After assessing the prey attractiveness (Eq. (6)), each predator modifies its motivation according to it. In order to maintain high motivation, the prey attractiveness should remain close to 1 (Eq. (9)).

(9) $${\rm new\ motivation = current\ motivation * prey\ attractiveness}$$

This value (“new motivation”), with range 0–1, is the probability of deciding to attack the focal prey. After the “Decision” is determined, it is internally stored until the actual action is conducted in the next time step.

#### Interaction frame: time step 2

“Predators-initial-act” procedure—Each predator conducts an action (attack or abandonment) according to the “Decision” made in the previous step. The probability of attack was the value of “new motivation” (Eq. (9)) at the end of the time step 1. If the predator decided not to attack, then it abandons the prey (which will revert to “resting” from the hitherto “approached” state) and will remain idle for the rest of the interaction frame. If the predator decided to attack, the predator–prey pair undergoes an initial attack event. In such an event, the prey (now in “attacked” instead of hitherto “approached” state) suffers a decrement in its survival chance (range 0–1). This effect is governed by the user-adjusted variable initial attack damage (range 0–1; Eq. (10)).

(10) $${\rm new\ survival\ chance = current\ survival\ chance * (1 - initial\ attack\ damage)}$$

“Predators-feel” procedure—After attacking a prey (which is now in “attacked” state), each predator receives taste information from handling the prey. Therefore, the predator could now update the unconditional stimulus value. In the current time step 2, the predator is performing its initial attack, so the information is updated from 0 to the repulsive taste (range 0–1; user-adjusted variable) of the prey (Eq. (11)). We assumed that the prey repulsive taste is a homogeneous characteristic across all prey, and there was no mutation or variation modeled. See “Discussion” for the implications of this assumption.

(11) $${\rm unconditional\ stimulus = repulsive\ taste}$$

“Prey-react” procedure—As a result of being attacked or abandoned by a predator, each prey is already in the “attacked” or “resting” state, and it reacts to that situation. The details of signal intensity change are identical to the “prey-react” procedure of the time step 1. In other words, based on its behavioral strategy (as described in the first section of “Methods”), the prey changes its signal intensity or remained in the current appearance.

“Prey-pay-cost” procedure—Each prey (either in its “resting” or “attacked” state) underwent a series of survival chance decrements, following the principles identical to the time step 1 (Eqs. (1), (2) and (3)). Especially, if the reaction involved switching of signal intensity to a different level, the prey paid the switching cost (Eq. (1)).

“Predators-observe” procedure—As it did in the previous time step 1, each predator experienced the conditional stimulus (Eq. (5)) again, but this time calculated with the current value of signal intensity displayed in step 2 (after predator initial attack—the “attacked” state of the prey).

“Predators-learn” procedure. The predator learns the association between the prey and the negative taste, and the speed of learning is determined by how striking the prey’s appearance (in its “attacked” state) is, how unpleasant the taste is, and how the program user set the global adjustment to all learning process. Technically speaking, each predator builds up aversive memory (Eqs. (12a) and (12b)) based on the newly updated conditional stimulus (Eq. (5)), unconditional stimulus (see the “predators-feel” procedure above), and a user-adjusted coefficient called learning speed.

(12a) $${\rm new\ aversive\ memory = current\ aversive\ memory + aversive\ memory\ increment}$$

(12b) $${\rm aversive\ memory\ increment = {\rm learning\ speed} * {\rm repulsive\ taste} * {\rm conditional\ stimulus}}$$

Aversive memory is any non-negative value with no upper bound, as explained in Eq. (7) and the accompanying text.

For the sake of simplicity, there was no memory decay modeled, and the predator population turnover was the only source of collective memory decrement. We made this assumption because taste aversions tend to have very high retention time (Steinert, Infurna & Spear, 1980; Elkins, 1984). It is possible for the users to modify the code and include memory decay if desired.

We allowed the speed of learning to be adjusted over very wide range. Similar to aversive memory, the user-adjustable learning speed also has no upper bound; very fast learning could induce substantial build-up of aversive memory even when both unconditional stimulus and conditional stimulus are fairly weak. For perspective, we used learning speed values from 0 to 1,000 in our study. Learning speed is an abstraction of the predator sensory and cognitive abilities as well as the environmental difficulties that might hinder acquisition of the aversion. For example, if Batesian mimics or other harmless yet similar-looking food sources are present in the habitat, the predator aversion learning would require longer time. The user might want to change the learning speed setting to form hypotheses involving such effects.

“Predators-analyze” procedure—Each predator uses the updated aversive memory, conditional stimulus, unconditional stimulus values to determine the prey attractiveness (Eq. (6)) again. The prey is still in its “attacked” state.

“Predators-decide” procedure—Each predator updates its motivation (Eq. (9)) based on the reassessed prey attractiveness. The new motivation value is the probability to attack the prey in the next time step 3. The prey is still in the “attacked” state.

#### Interaction frame: time step 3

“Predators-subsequent-act” procedure. It is almost identical to the “predators-initial-act” procedure in the previous time step. This time its action is either a subsequent attack event (prey stays in the “attacked” state) or an abandonment (prey reverts to “resting” state), and the prey survival chance is affected by subsequent attack damage (range 0–1), another user-adjusted variable (Eq. (13)).

(13) $${\rm new\ survival\ chance = current\ survival\ chance * (1 - subsequent\ attack\ damage)}$$

“Predators-feel” procedure. Each attacking predator receives the updated unconditional stimulus as it did in the previous time step. Since this is a subsequent attack, the information would not be different from what it felt in the initial attack. Each prey is either in “attacked” or “resting” state.

“Prey-react” procedure—Each prey reacts to the subsequent attack in the same manner it did in the previous time step 1 and 2, following its behavioral strategy (see the first section of “Methods”). Depending on whether the subsequent attack was executed, the prey is currently in either “attacked” or “resting” state.

Note that the predator is going to leave the site afterward regardless of the prey response, as the simulation will not give any more subsequent attack event. Therefore, in some situations, the prey would gain little benefit from reacting to this last attack from the predator. However, we assumed that the prey cannot know if the predator interaction is coming to an end, and that it will unconditionally display the signal as long as its behavioral strategy assigns a response to attack.

In other situations, strongly reacting until the end of the attack (and thus giving prolonged stimulus to help predator learning) may increase prey fitness, if the prey or its offspring is likely to encounter that identical predator individual again in the future.

“Prey-pay-cost” procedure—Each prey (in either “attacked” or “resting” state) suffers a drop in survival chance, as it did in the previous time step 1 and 2 (Eqs. (1), (2) and (3)).

“Predators-observe” procedure—Each predator gathers the conditional stimulus (Eq. (5)) based on the updated signal intensity information (from the prey in “attacked” state), as it did in the previous time step 1 and 2.

“Predators-learn” procedure—Each predator increments its aversive memory (Eqs. (12a) and (12b)) with the updated conditional (Eq. (5)) and unconditional (see the “predators-feel” procedure) stimuli as well as the user-adjusted learning speed. Each prey is still in either “attacked” or “resting” state.

At the end of an interaction frame, all prey individuals reverts to the “resting” state.

#### Reproduction stage

“Prey-selectively-die” procedure—Each prey survives or dies according to the final value of its survival chance.

“Prey-lay” procedure—One of the surviving prey individuals is randomly chosen to generate a clone that will live in the next generation. The process is repeated until the carrying capacity (180 individuals in our case) of the next generation is all filled.

“All-prey-die” procedure—Every prey of the current generation dies out, only leaving the next-generation population.

“Prevent-extinction” procedure—In our model, a given prey phenotype could not undergo a complete extinction. As a result of the prior procedures, a prey behavioral strategy could have temporarily gone to extinction at the previous procedure. Then, the program forcefully rescues the situation by generating a new prey individual with the extinct trait at the expense of a random existing individual. It was a design choice to prevent premature fixation and improve the robustness of the model outcome. With this procedure, the model could maintain the variation without any mutation or recombination; it helped us to minimize quantitative assumptions that are not directly related to our question.

“Predators-turnover” procedure—A number of naïve predators enter the world and replace some of the old predators. The user-adjusted variable predator turnover rate determines the proportion of the predators that are replaced by fresh ones, and the turnover occurs at the onset of the next prey generation. The new predators joining the model have zero aversive memory.

#### Final outcome: the winning strategy

After 500 prey generations we determined the outcome of the simulation. The winning strategy was defined as the most abundant behavioral strategy among prey.

### The settings used for this article

*ApoSim* has many user-adjusted settings and variables (Table 2), and it was impractical to explore all the possible combinations in one focused study. Instead, we focused here on a subset of questions arising from the existing literature as outlined in the Introduction. We asked how several types of costs that prey incurs affect the evolutionary outcome, and how does the outcome depend on the learning speed and the influx of naïve predators into the system.

**Table 2:** The list of model parameters and the values used in this paper.

Parameter	Value(s)
Controlled variables^†^	
World size	14
Generation length	50
Prey frequency	92%
Predator frequency	23%
Low-signal (L)	0.3
High-signal (H)	1
Initial attack damage	0.1
Final attack damage	0.4
Basal cognitive cue	0.1
Repulsive taste	0.1
Behavioral strategies	(NNN, NNL, NNH, NLL, NHH, LLL, LLH, LHH, HHH)
Independent variables	
Switching cost^††^	“none” (0), “moderate” (0.0005), “high” (0.016)
Switchability maintenance cost^††^	“none” (0), “moderate” (2.03E−4), “high” (0.00340)
Basal detectability	0.05, 0.15, 0.4, 0.8
Predator turnover	0.01, 0.25, 0.7, 1
Learning speed	0, 0.01, 0.03, 0.1, 0.3, 1, 3, 10, 30, 100, 300, 1000
Signal penalty	0.13, 0.1105—ratio 0.85, geometric sequence—5.32E−5, 4.52E−5, as well as 0

**Notes:**

† Not all controlled variables are explained in the main text, as some of them are not relevant to the questions asked in this paper. For the full description of all simulation parameters, please see Supplemental Materials.

†† For the purpose of this paper, the two variables were treated as one set under the name “penalty of switchable signal”, and they changed simultaneously between three states: none, moderate, or high.

We decided to vary only five variables, as five-dimensional data would be close to the limit of meaningful visualization. We chose three variables relevant to general costs of signaling: basal detectability, signaling penalty, and penalty of switchable signals (which was composed of switching cost and switchability maintenance cost). We used two independent variables representing the properties of the predators: predator turnover and learning speed. Note that technically we varied six variables, but switching cost and switchability maintenance cost were similar in nature, and we decided to co-vary them assuming positive correlation (which may not be true in some natural systems).

The combination of these five independent variables led to 29,376 different conditions, and we repeated the runs three times in each condition. This resulted in 88,128 outcomes in total. Besides these five independent variables, we treated every other user-adjustable variable as a controlled variable; we gave a fixed, reasonable value for each of them (Table 2). We want to emphasize that a future user of this software can choose completely different sets of independent variables.

Due to this incomplete search of the parameter space, our model result should be interpreted with caution. Our choice of control variables (Table 2) is supported by parameter-bracketing results (Fig. S3); however, it is still a set of arbitrary assumptions that should not be directly related to quantitative measurements from the real world.

Among the variables shown in Table 2, the list of behavioral strategies needs more clarification. As we mentioned in the first section of Methods, one of our core ideas is that the user can describe a variety of behavioral strategies in the three-letter notation.

Out of 27 possible combinations, we chose nine strategies for this study based on the following assumptions. First, the prey can have only one switching opportunity at maximum. Therefore, the strategy can be either one of the pre-attack or post-attack switching, but not both. This was to aid visualization by making the competitive alternatives highly contrasting; compromised intermediates can be difficult to plot and describe. Second, we assumed that increasing threat level can only be associated to increasing levels of signal intensity; the prey should stay at the current level or switch to a higher signal when freshly “approached” or “attacked.” In the current version of the software, the predator detection check is only done once. This limitation made it useless to hide after initial interaction, because such behavior cannot alter the outcome of that encounter. With these two restraints, the possible combination of strategies reduced to nine as seen in Table 2.

## Results

**Figure 2:**
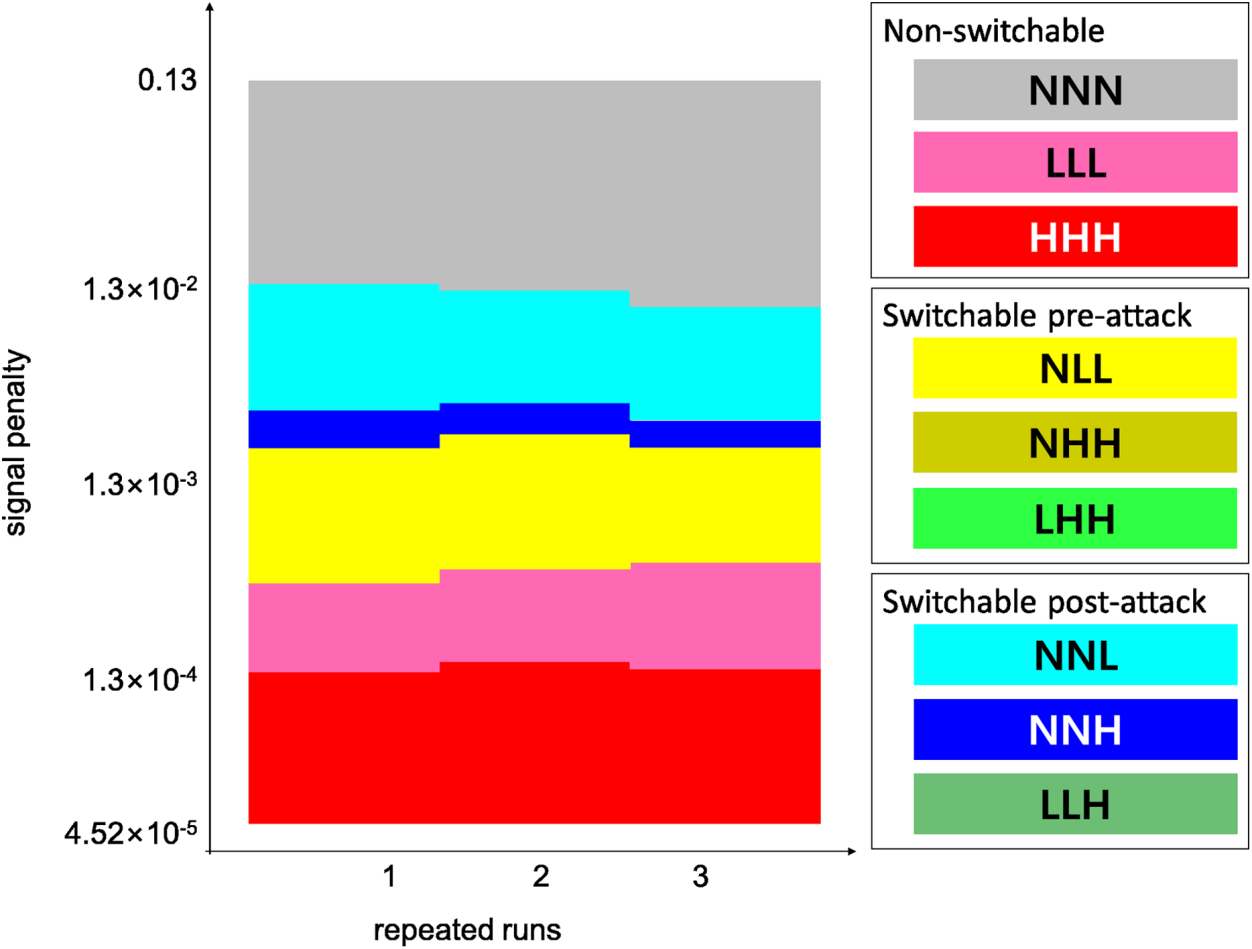
An example mini-plot of the model results and the color codes for the behavioral strategies. The prey behavioral strategy that won each run is depicted according to the color code. For the three-letter annotation of the behavioral strategies, see the first section of “Methods”. The vertical axis is the signal penalty value used in each run. There were three repeated runs for a given combination of conditions, and these repeats appear along the horizontal axis. Therefore, a mini-plot visualizes the result of 153 runs in total, with 51 signal penalty values and three repeats. For more detailed information about the run parameters, see Table 2. Mini-plots like the one shown here are the building blocks that constitute the meta-plots in Fig. 3.

**Figure 3:**
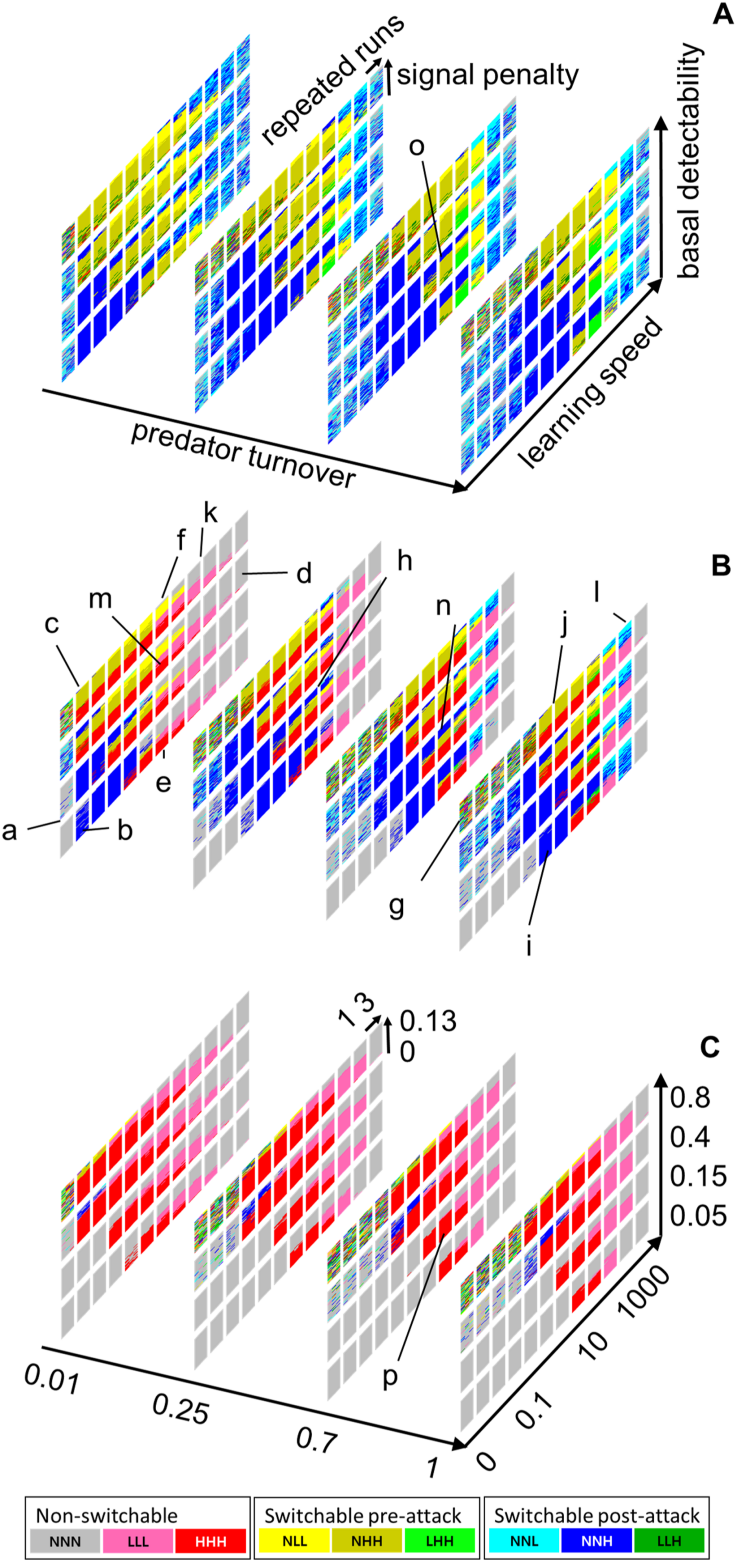
Model result meta-plots. The winning behavioral strategies over the entire range of the tested parameters given in Table 2. For the color code and the interpretation of each mini-plot, see Fig. 2. The *x*-axis, the one that runs from left to right, is the predator learning speed. The *y*-axis is the predator turnover rate. The *z*-axis is the basal detectability of the prey. For more detailed information about each variable, see the “Methods” section. (A) The switching cost and the switchability maintenance cost are both none. (B) there are moderate costs for both. (C) the costs are prohibitively high. For more detail about the three levels of penalty of switchable signals, see Table 2. Note that in order to avoid the visual clutter in the three-dimensional view, we labeled A–C with different sets of information; the names of the axes, the regions of interest in lowercase letters, and the values of axes are separately given in A–C, respectively. However, all notations are applicable to all three subpanels. Regions of interest indicated by small letters (a–p) are referenced in the main text of the “Results” and “Discussion” sections.

Two-dimensional mini-plots (Fig. 2) are combined in three-dimensional meta-plots (Fig. 3) that contain the outcomes of 88,128 model runs. Here, we briefly describe the resulting Fig. 3, highlighting comparisons among the figure elements that later in Discussion will be invoked again. From now on, we will regard Fig. 3B, for the moderate switching cost and switchability maintenance cost, as the standard result analyzed in detail. We will then describe differences in Fig. 3A or 3C compared to Fig. 3B.

In Fig. 3B, NNH, NHH, or HHH behavioral strategies generally appeared in the moderate learning speed range. Conditions with extremely slow-learning predators favored NNN, the none-signal strategy (e.g., “a” of Fig. 3B). As the learning speed increased, strategies with high signal intensity began to appear (e.g., the transition from “a” to “b” of Fig. 3B). The opposite extreme condition with fast-learning predators also favored NNN (e.g., “d” of Fig. 3B). There was a tendency of smooth tapering from high- to low- and finally to no-signal strategies as the learning speed increased (e.g., the transition from “e” and “f” columns to “d” column in Fig. 3B).

Conditions with higher basal detectability and higher learning speed selected the pre-attack or permanent signaling strategies, while lower basal detectability and lower learning speed favored post-attack strategies (e.g., contrasts between “b,” “c” and “f” in Fig. 3B). If the basal detectability was extremely high while the predator learning speed was extremely slow, then there was no stable winner, and the outcome was mostly random (e.g., “g” of Fig. 3B).

If learning speed and basal detectability were both in the moderate range, the signaling penalty dominated the performance of behavioral strategies. The lower signaling penalty led to stabilization of permanent signals; higher signaling penalty favored post-attack signals; pre-attack signals won in the middle ground (e.g., “h” of Fig. 3B).

Finally, if the predator turnover was high, the influence on the outcome appeared mostly identical to the slowing down of the effective learning speed. For example, in Fig. 3B, the shift from “e” to “b” (decrease in learning speed) was similar to the shift from “e” to “i” (increase in predator turnover.) However, when the learning speed was high, the predator turnover rate was a determining factor for the emergence of the post-attack signals: if the turnover was low, higher learning speed conditions mostly selected permanent or no signals strategies (e.g., “k” of Fig. 3B); if the turnover was high, post-attack signals were also selected (e.g., “l” of Fig. 3B).

Figure 3A shows the corresponding set of results with no fixed or per-use costs in switching signals (i.e., switching cost = 0 and switchability maintenance cost = 0; Table 2). In this condition, permanent signal strategies disappeared almost entirely, and the switching strategies replaced them. Another difference is that the post-attack switching strategies became more common in normally unfavorable, extreme conditions.

Figure 3C shows results for situation in which the switching phenotypes pay very high costs (i.e., switching cost = 0.016 and switchability maintenance cost = 0.000255; Table 2). The figure shows that the no-signal strategies replaced most of the switching strategies. The switching phenotypes survived only in the narrow zone of moderate learning speed, high basal detectability, and extremely high signaling penalty.

To assess the robustness of the result, we have run simulations equivalent to Fig. 3 with different levels of control variables (Fig. S3). Readers should be aware that however reasonable they are, our choices of control variables and their values are strictly arbitrary. Furthermore, as apparent in the Methods, the model structure is an abstract and simplified simulation, not a faithful reconstruction of a complex natural system. Therefore, the numerical values form the model should be interpreted as generic tendencies, rather than exact quantitative predictions of relationships that could be empirically discoverable in the real natural systems.

## Discussion

The results of the model (Fig. 3) help our understanding of possible evolutionary transitions between various forms of aposematic adaptations in prey. For instance, a defended prey may undergo evolutionary transitions from NNN (non-signaling) to NHH or NNH (examples of switchable aposematic signals), from NNN to LLL or HHH (permanent aposematism), or from HHH to NNH or NNN. Our model visualizes how such evolutionary transitions may be driven by changes in ecology of predators and prey. Here, we will first discuss in details the conditions that favor specific anti-predatory adaptations by focusing separately on each of the five independent variables (Table 3). Then we will discuss possible evolutionary scenarios towards switchable aposematic signals. Finally, we will also look closer at some of the existing and novel hypotheses about the evolution of various forms of aposematism and how the model may contribute to understanding of their evolution. To achieve clarity and precision of our presentation, here we refer by number to relevant figures and tables in the “Results”.

**Table 3:** The independent variables used in the paper and the corresponding natural examples.

Variables	Short definition	Examples (low level)	Examples (high level)
Costs to prey			
Basal detectability	Basal discovery chance when not giving a signal	Dead leaf mantis nearly impossible to discover when not doing threat display	Stotting ungulates their size and habitat often make them highly noticeable even when not leaping
Signal penalty	Inherent cost associated to higher level of signal	Skunk body stripes there is little evidence of disadvantage for more striking bands	Black widow spider the red dorsal mark is selected to be moderate because it can be seen by prey insects (Brandley, Johnson & Johnsen, 2016)
Switching cost	Cost paid per each switching behavior	Color under wings half a wingbeat to expose pigmentation is very cheap compared to regular flight	Fire-bellied toad unkenreflex the complete flip of the body is a big, energetic movement that may hinder many other useful behavior
Switchability maintenance cost	Cost of maintaining the ability to switch signals	Color under wings if flight is maintained anyway, there is almost no additional cost to switchability	Rattlesnake rattle fragile structure that is kept lifted in locomotion; the tail tip cannot be used in versatile activities like other snakes
Properties of predators			
Learning speed	Coefficient of aversive memory buildup	Mantis-milkweed bug many intermediate stages before reaching complete aversion (Gelperin, 1968)	Primate-snake aversion acquired quickly and intensely (Ohman & Mineka, 2003)
Predator turnover	Proportion of naïve predators entering per prey generation	Blue jay-monarch butterfly every year only a fraction of the population needs to learn about cardenolide	Mantis-milkweed bug probably the whole annual population needs to freshly learn the aversion (Gelperin, 1968)

### Predator learning and population turnover

The results are consistent with the existing theoretical coverage of the effects of predator learning and forgetting on prey aposematic signals (Speed, 2001). For example, some of our model runs included predators whose learning speed is so extremely slow that the aposematic signals become useless. This condition is seen in “a” of Fig. 3B. Increase in the predator learning speed eventually leads to the relatively abrupt appearance of the strongest possible signals (H), be it switchable (Figs. 3A and 3B) or not (Fig. 3C; here the permanent signals are less costly). One such pattern is visible in the transitions from “a” to “b” in Fig. 3B. This suggests that there may be a threshold of predator learning speed, below which aposematism (switchable or permanent) cannot outcompete the cryptic forms. This is because the prey cannot give infinitely effective signal while the predator learning speed can reach near zero; below some point of learning speed the prey should fall back to crypticity instead of investing more into the signal. When the predator learning speed is barely above this crypticity-aposematism threshold, strong signals (H) are observed (e.g., “b” or “c” of Fig. 3B) because high signal strength is needed to cause efficient learning in the relatively slow learning predators.

As seen in Fig. 3B, the crypticity-aposematism threshold is highly dependent on the predator turnover and basal detectability. Predator turnover determines the benefit of aposematism, as it determines the residence time of the educated predators; basal detectability, the baseline chance of being discovered, determines the benefit of crypticity.

Slightly above the crypticity-aposematism threshold, the post-attack aposematism frequently dominated the runs (e.g., from “a” to “b” in Fig. 3B). When predators learn slowly there is a higher chance of encountering a predator with little knowledge, and it is more likely to be attacked upon discovery. Under this condition, post-attack strategies, which minimize the risk of detection, should be favored if switching-related costs are not too high (Figs. 3A and 3B).

On the other extreme of learning speed, our model shows an alternative situation. Under very high learning speed (e.g., “d” of Fig. 3B), NNN strategies are the most successful ones. In our model, predators can learn from the prey basal cognitive cue even when no signal is given from the prey. If learning speed is high enough, this basal learning can grant adequate protection. Hence, in this situation the basal cognitive cue fulfills aposematic function; it should be noted that the phenotype NNN is actually protected by aversive learning unlike the situation at the slower extreme of learning speed. It is possible that some fast-learning predators may indeed not need any auxiliary signal from the prey in order to recognize it as defended. For instance, it has been demonstrated that oriental tits can learn the basic, inconspicuous morphological features of an insect as cues of unprofitability, albeit at a slower rate compared to learning of a typical aposematic signal (Kang et al., 2016). This signal-free, cue-based aposematism is expected to have a widespread significance in evolution of many predator-prey systems, because there exist a number of cue-based protective mimicry (De Solan & Aubier, 2019).

The transition toward this “inconspicuous” form of aposematism appears to be more gradual (e.g., from “m” to “d” in Fig. 3B) compared to the relatively abrupt transition between NNN and signaling strategies (e.g., from “a” to “b” in Fig. 3B) at the slower learning speeds, which we have already discussed. This effect of a very high learning rate could be inferred from earlier models (Speed, 2001; Puurtinen & Kaitala, 2006). Especially, the gradual transition toward weaker conspicuousness fits well to the situation described in seasonal predator psychology studies (Endler & Mappes, 2004; Mappes et al., 2014).

The effect of the decreased predator population turnover largely corresponds to the effect of increased learning speed. As a result, the entire three-dimensional plot is compressed/shifted to the right side (requiring higher learning speed) as predator turnover increases. This is because the influx of naive predators hinders the collective memory build-up in the predator population, and the average predator behavior for a given prey becomes similar to what can be expected in slower-learning but lower-turnover predator population.

However, the high predator turnover leads to a unique outcome with the very fast-learning predators that can associate prey defense with the prey basal cognitive cue. As apparent in “l” of Fig. 3B, which can be contrasted to “k” of the same figure, high predator turnover favors the evolution of weak post-attack aposematism when the predator learning speed is sufficiently fast. In this scenario, the predator knowledge level is highly variable (because of the high turnover), and the prey cannot know if the approaching predator is a naive or experienced one. Therefore, the post-attack signal, which would be useless if the predator population was full of well-educated predators, is still needed to educate and deter the fresher portion of the predator population.

The mix of experienced and naïve predators has been a popular topic in studies of permanent aposematism (Lindstrom et al., 1999; Speed, 2001; Svadova et al., 2009). Especially, the studies on the seasonal variation of predator knowledge (Endler & Mappes, 2004; Mappes et al., 2014) provide highly advanced analyses about the continuous influx of naïve predators and the need to repeatedly educate them. It was suggested that the post-attack display of the spotted lanternfly, *Lycorma delicatula*, is an adaptation to this circumstance (Kang, Lee & Jablonski, 2011; Kang et al., 2016). The results of our simulations indeed confirm that the weak post-attack switchable aposematism is actually the best strategy to deal with the mix of naïve and experienced predators of high learning speed.

### Basal detectability

Empirical and theoretical studies about deimatism (startle display) and post-attack defense have already discussed the general effect of the basal detectability (Broom, Higginson & Ruxton, 2010; Willink et al., 2013; Umbers, Lehtonen & Mappes, 2015). However, to our knowledge, there is no currently available literature that focuses on evolution of switchable aposematism while overviewing the importance of the basal detectability along with other driving forces.

Our results are consistent with the general prediction that low basal detectability would favor the post-attack aposematism (e.g., panel “b” in Fig. 3B) rather than the pre-attack or the permanent aposematic signals (e.g., panel “c” in Fig. 3B). Initiation of an attack by a predator is a clear indication that the prey has been spotted. But before that moment of attack there is always a chance that the approaching predator has not detected the prey, especially for prey with low basal detectability. In this situation, it would not be beneficial to the prey to use a pre-attack display because such a behavior would reveal its presence to the predator, who otherwise is unlikely to be aware of its location. Similarly, the prey with permanent aposematic signals would entirely lose the opportunity to benefit from the potential crypticity. On the other hand, the post-attack signals are only emitted when the predator has already detected the prey and decided to attack (Broom, Higginson & Ruxton, 2010). In such a scenario, giving post-attack aposematic signal cannot increase the risk of detection, as it is already detected. Hence, if the prey can energetically and ecologically afford the brief display of the signal, the post-attack switch-on can only help the prey, not harm it (unless the predator is more intrigued by the signal received during the attack). Therefore, post-attack signals can be a viable strategy in the conditions that are not favorable to pre-attack or permanent alternatives.

Higher basal detectability, on the other hand, effectively lowers the opportunity cost of aposematism. As an approaching predator is likely to detect the conspicuous prey anyway, the pre-attack or permanent aposematism would not bring much additional risk. As discussed before, the low learning speed is a condition where post-attack signals generally prevail. However, the high basal detectability and the reduced opportunity cost can bring the balance toward pre-attack/permanent aposematism (transition from “b” to “c” of Fig. 3B). If the additional risk is negligible, pre-attack or permanent aposematism offer faster memory retrieval which grants the prey further protection.

If the predator learning speed is too low in this condition, then the signal is void of both risk and benefit - it may not significantly add to either detection risk or predator deterrence. In this situation, the selection pressure on aposematism-crypticity axis is largely absent, and no specific strategy would be clearly favored (e.g., “g” of Fig. 3B) under our set of assumptions.

### Costs of signals

The general effect of the various types of costs on the evolution of post and pre- attack defenses have been analyzed earlier (Broom, Higginson & Ruxton, 2010). However, to our knowledge, there had been no overarching theoretical perspective that shows the full implications of these costs when faced with learning-capable predator population. Our results provide a solid background to discuss the interactions between costs of a signal and costs of its switchability pertaining to the evolution of aposematism.

The signaling penalty is a value that represents the energetic cost of the signal as well as the exposure to the potentially signal-unfriendly environment (Table 3). If other variables have moderate values, the increase in signaling penalty leads from permanent to pre-attack, and to post-attack aposematism (as seen in “h” of Fig. 3B and other similar mini-plots). As the signal is given for shorter and shorter duration along the above sequence, it is the logical order to constrain the increasing signaling penalty cost into an affordable range. However, as seen in most of the other mini-plots in Fig. 3B, one should note that if other variables do not favor certain forms of aposematism, the signaling penalty alone—even when extremely high or low—is insufficient to promote all possible types of aposematism.

Figure 3A shows the model outcomes when switching cost and switchability maintenance cost (together representing the “penalty of switchable signals”) are absent while signaling penalty is present. In this condition, the permanent signals would be generally inferior to the switchable alternatives because of the longer duration of unnecessary exposure. Without any additional cost, the switchability provides finer control over the signal intensity fitting the circumstances. Therefore, the permanent aposematism present in Fig. 3B mostly disappeared in Fig. 3A.

Though less pronounced than in the permanent signals, some difference in the post-attack signal is also visible in Fig. 3A; the post-attack signals are more likely to stabilize in signal-unfriendly extremes. Being switchable, post-attack signals have two types of costs—the signaling penalty (cost associated to the signal intensity) and the switching-related costs (the fixed switchability maintenance cost and the per-use switching cost). Considering that the post-attack signal is presented only after a predator attack, the signaling penalty would be largely avoidable during the most of prey lifetime. Therefore, the reduction of switching-related cost could substantially alter the cost-benefit balance of this strategy, which can be seen from the difference between Figs. 3A and 3B.

Figure 3C shows the other end of the spectrum. It depicts the model outcome when the signal-switching behavior and the maintenance of switchability are very costly. Naturally, almost all switchable signals disappeared, and permanent aposematism became more common. In comparison to Fig. 3B, one can see that the pre-attack signals mostly changed to the permanent signals while post-attack signals generally reverted to permanent none-color strategy. Permanent signal is a good substitute for pre-attack signal except for the lengthened display duration; in predator deterrence, they serve essentially the same purpose. On the other hand, the stability of the post-attack aposematism is mostly due to the utilization of crypticity while not being attacked. Therefore, if switchable signals are not an option due to high costs, the post-attack strategy is replaced with the no-signal strategy that maintains the benefits of crypticity.

### Conditions for evolution of switchable signals

**Figure 4:**
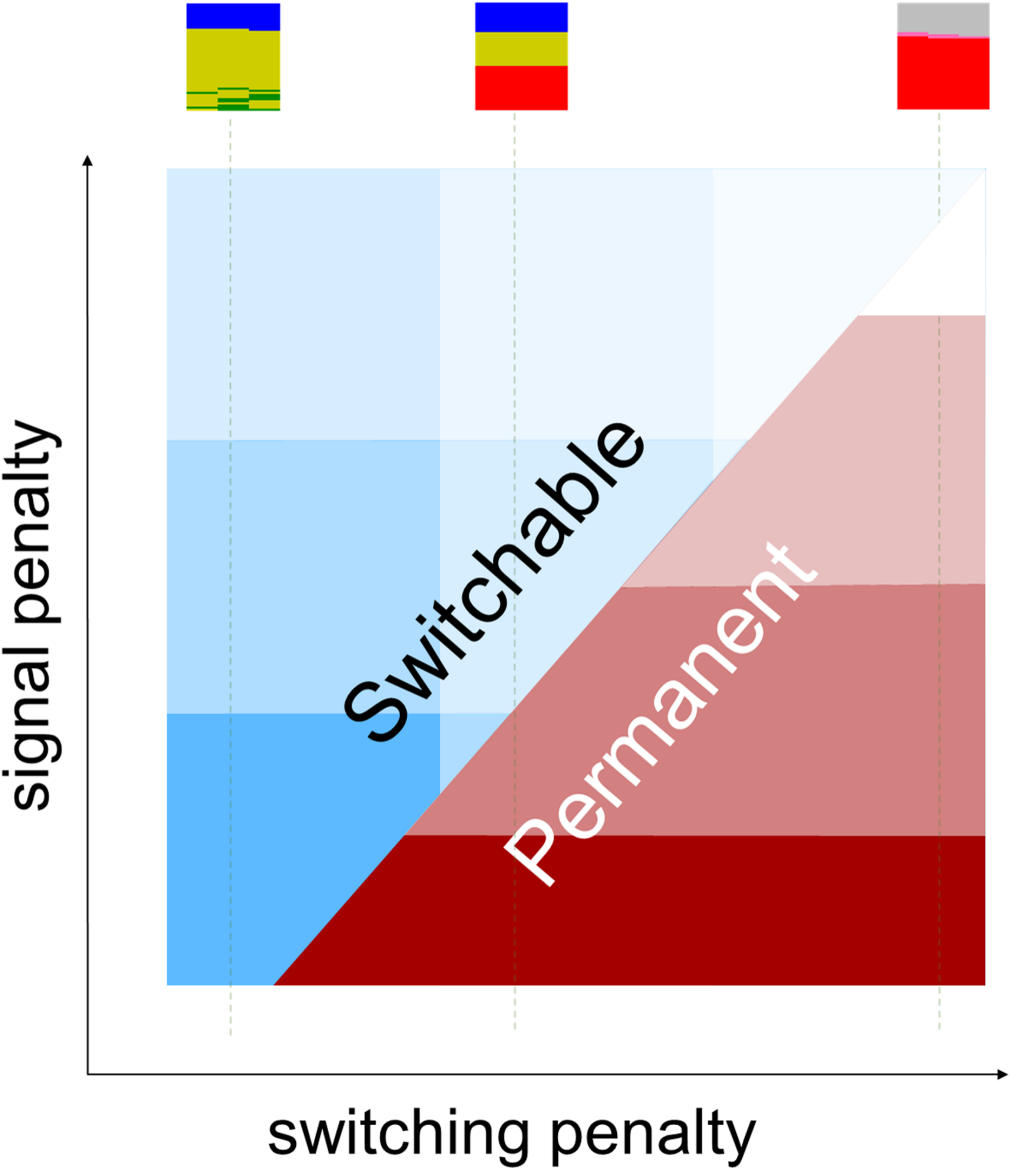
A two-dimensional diagram for evolutionary stability of permanent versus switchable aposematism. This is an evolutionary “phase diagram” to abstractly express the stability of the two modes of aposematism. The horizontal axis is the signal penalty, and the vertical axis is the switching penalty (a collective representation of both per-use switching cost and the fixed switchability maintenance cost). The reddish-brown color represents the strength of permanent signals that are expected to be stable in the given condition. The blue color represents the strength of the switchable signals in general, without distinguishing pre- from post-attack switching. Both colors fade as the costs increase indicating that neither permanent nor switchable displays are likely to evolve for extremely high values of both costs. Inlets are mini-plots “n,” “o,” and “p” of Fig. 3, reproduced as examples of three switching penalty levels. For the color codes, axes and ranges of the mini-plots, please see Figs. 2 and 3.

Based on the model results, we hypothesize that the relative values of the two types of cost, the signaling penalty and the penalty of switching, predict the evolution of switchable or permanent signals (Fig. 4). As the costs of switchable signals increase from Figs. 3A–3C, the switchability-favoring range of signaling penalty narrows down (e.g., from the whole range in panel “o” in Fig. 3A to none in in panel “p”). We deliberately configured the software to exclude any startling (“deimatic”) or learning facilitation effects of the switched signals. In the setup used in this paper, the strength of the signal being switched on is the only relevant factor in predator psychology; the fact that it was switched does not have any effect except that it imposes some cost to the prey. This decision helped us to study the cost-saving aspects of switchable aposematism without further complications. However, this assumption is far from reality (Umbers, Lehtonen & Mappes, 2015; Kang et al., 2016; Ruxton et al., 2018), and the model predicts that the switchable aposematism would be more favored if startle and facilitation effects could provide survival advantages (Fig. S1).

### Visualizing hypothetical evolutionary pathways leading to aposematism

**Figure 5:**
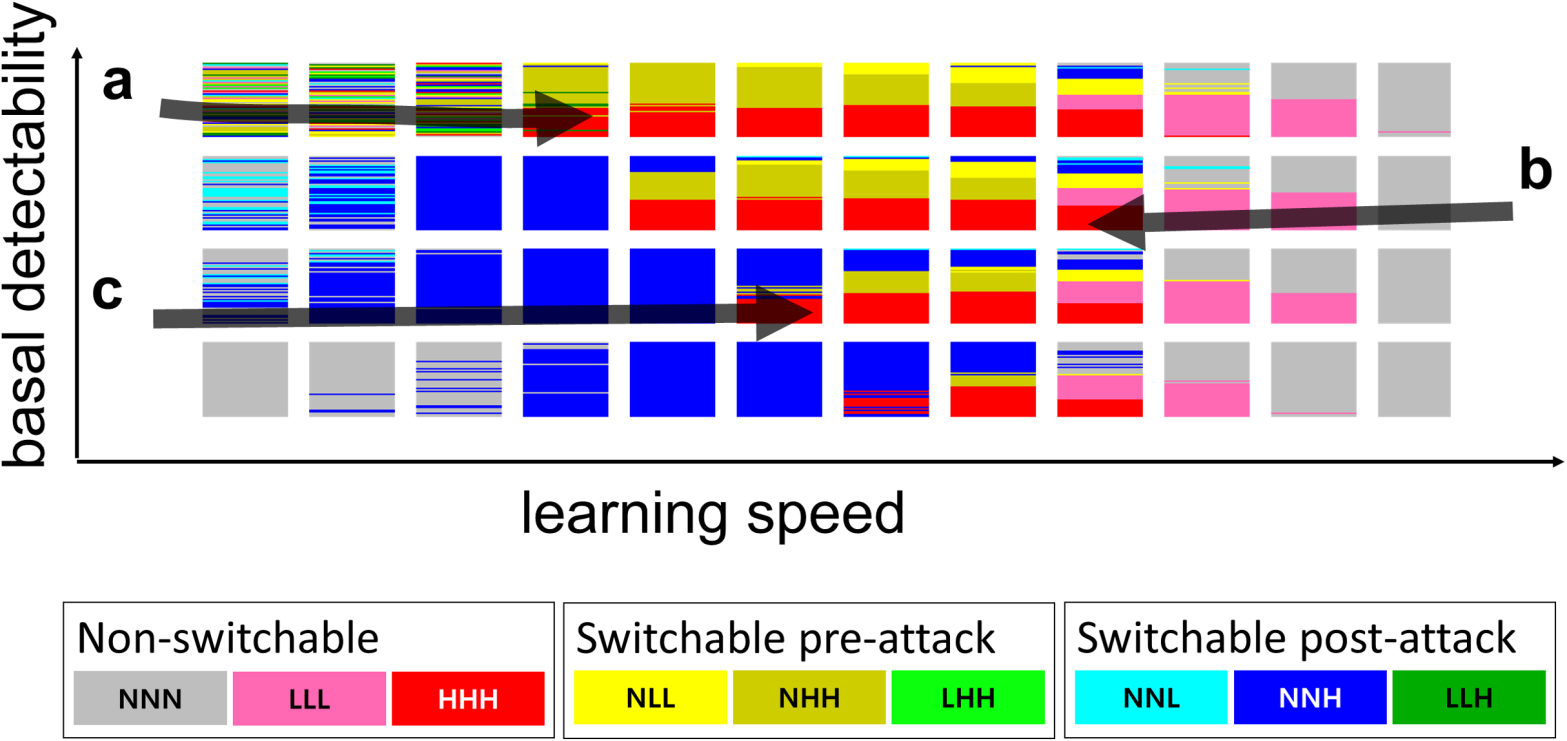
Three hypothetical pathways towards aposematism. A portion of the model outcome meta-plot in Fig. 3 is shown again for explanatory purpose. The labeled arrows (a–c) illustrate the hypothetical pathways, and each is referenced in the main text. These are examples of how the model can be used to discuss hypothetical evolutionary pathways between various forms of (non-)aposematism. Hypothetical mechanisms and ecological conditions critical along such pathways can be identified using the software.

Although our model is not designed to directly imitate predator-prey coevolution, its results (such as Fig. 3) can provide a useful aid to contemplate the evolutionary effect of changes in environment and in predator ecological guild. Fig. 5 shows how such changes may mediate a variety of possible evolutionary pathways to different forms of aposematism in the defended (unprofitable) prey.

Scenario “a”: highly detectable prey invokes learning in predators—the path “a” of Fig. 5 represents a hypothetical scenario starting from non-cryptic and non-aposematic prey phenotypes. The ancestral non-crypticity (high basal detectability) could have emerged by chance, or due to selective processes other than aposematic signaling. Examples of such conditions include sexual selection and conspicuous behaviors such as flight. Our model predicts that such traits would affect the existing predation pressure very little if basal detectability is high and the learning speed is slow (see Basal Detectability section of “Discussion”). Once the defended prey has acquired those non-cryptic unique characteristics that are different from the undefended ones in the same ecosystem, a selection pressure to distinguish them may apply to the predators. This can lead to an increase in predator learning speed, which may in turn favor signaling strategies in prey, thereby initiating a true aposematic interaction.

This scenario may be considered in sexual selection context (Maan & Cummings, 2009; Crothers, Gering & Cummings, 2011; Ruxton et al., 2018), because it provides a straightforward condition that can lead to highly conspicuous appearances before aposematic evolution occurs. Another important example regarding this pathway is the mobility benefit (Speed, Brockhurst & Ruxton, 2010). If the prey is already conspicuously mobile due to the need in resource collection, this is a condition that highly favors the evolution of aposematism. Our visualization in “a” of Fig. 5 is consistent with those two scenarios and expands them with the insights gained from other related variables (Fig. 3).

Scenario “b”: quick-learning predators may provide a starting ground for evolution of aposematism—path “b” of Fig. 5 illustrates a hypothesis that the ancestral predators have very high learning capability. In this scenario, the basal cognitive cue—the minimal uniqueness in body shape and behavior that arose from prey ecology—has been already performing an antipredatory aposematic function (see Predator learning and population turnover above). However, the variation in predator intelligence could have demanded some slight ‘nudge’ to help the defended prey in being correctly discriminated. Once the prey evolves this minimal cognitive aid that increases its survival, then this frees up the predators from the burden of cognitive and behavioral investment that enabled the initial identification. This degradation of learning capabilities then could have begun the down-spiral toward highly conspicuous aposematic systems. In the final stages, as in the previous scenario, the relative ratio between signaling and switching penalty determines whether permanent or switchable aposematism is established.

Alternatively, the path “b” is also consistent with a scenario that does not assume co-evolution but simply represents a change of ecological guild of predators to which the prey is exposed: from extremely fast learners to intermediately fast learning predators. One can easily imagine that a prey invading new habitats may occasionally experience such changes. Similarly, a sudden or gradual change of predators’ guild composition may shift the prey conditions from facing the “very fast learning” predators to facing “moderately fast learning” predators. By considering such ecologically driven gradual evolution of prey signals, the scenarios along pathway “b” directly provide alternative solutions to the controversy surrounding the possibility of gradual development of aposematism through co-evolution (Lindstrom et al., 1999; Endler & Mappes, 2004; Ruxton et al., 2018).

Scenario “c”: post-attack switchable aposematism as ancestral form of aposematism—It is reasonable that a prey at the post-attack stage would be more easily identified than at the pre-attack stage. After being attacked the prey would attempt to struggle or flee, revealing the prey’s various body parts, behavioral peculiarities, and other information. Even if the predators have not used this information yet, the prey might have been already under selection to intensify this post-attack behaviors in order to increase survival through escape. This may lead to more and more unique and conspicuous prey reactions, not because the conspicuousness was needed but as a byproduct of selection toward effective escape behaviors. It is feasible to assume that this intensification of post-attack behavior involves increase in its memorability to the predator. The higher memorability can effectively increase the learning speed, shifting the conditions from favoring “none-signal” (gray shaded panel in Fig. 5) to those favoring post-attack signal (blue panels). Alternatively, the predators might evolve better cognitive ability (learning speed) to recognize the post-attack behavior of the defended prey because it may increase their foraging efficiency. Either way or both, our model predicts that the shift in the learning speed would lead to a new selection regime that will drive strong post-attack aposematism.

An alternative version of the above process may involve a shift from ecological conditions favoring no signaling (gray panels in Fig. 3) to conditions favoring post-attack aposematism (blue panels) due to a decrease in the influx of naïve predators (decrease in the predator turnover rates). This can be seen in Fig. 3B where panels in the lower left corner (low learning speed, low basal detectability) change from gray (no signals) to blue (post-attack signals) as the predator turnover decreases.

After post-attack aposematism is established for any reason, the pressure to maintain prey crypticity (i.e., low basal detectability) can be relieved if the strategy provides sufficient protection. In this condition the prey may become more easily detectable, and this trend may be associated with the increase in prey memorability through the heightened basal cognitive cue and the encounter frequency. The increased memorability is a boost to learning speed by itself, and it may also trigger natural selection for faster learning; the cognitive investment may now bring more benefits in foraging efficiency. The increased learning speed may create a situation when the post-attack is no longer the evolutionarily winning strategy. Instead, depending on the ratio of switching penalty to signaling penalty, the pre-attack or permanent aposematism would be favored. In summary, this scenario shows that the post-attack aposematism could open evolutionary pathways to other forms of aposematism. This hypothesis is similar to an existing theory about evolution of aposematism in physically defended animals (Speed & Ruxton, 2005) except that our focus is on behavioral reactions rather than on the physical devices.

### Model assumptions and limitations

The above discussions illustrate how our model may contribute to research on diversity of aposematic strategies. However, in order to draw proper generalizations and use the model to answer new questions, one needs to understand the model’s assumptions and limitations.

First, our model does not feature any coevolution, neither between species nor between traits. Predators can only learn and do not evolve over generations; the prey behavioral strategy is the only trait that has variation for natural selection. In nature, one can expect that there would be predator-prey coevolution as well as the coevolution between behavior, morphology, and physiology. However, proper modeling of such evolutionary interactions would require a number of quantitative assumptions that would limit the applicability and usability of the software. Considering that the signaling behavioral strategy is already a multi-dimensional variable, we found that the further complexity in the modeled world give little benefit while immensely interfering with the visualization, hypothesis formation and the interpretation of reality using the model. Instead, by keeping constant the components that are reasonably expected to evolve slower than prey behavioral strategy, we were able to obtain a complete view across a wide range of conditions. Our primary goal was to generate clear predictions about optimal prey behavioral strategies in a given combination of environmental variables and predator phenotypic and population traits. We realized that modeling coevolution, albeit intriguing, could complicate the accomplishment of this goal.

One omitted variable, however, deserves a more detailed discussion: prey defense. Our model treats prey defense as a given fixed value for each prey individual (repulsive taste), not allowing for variation among prey individuals. It could be seen as an unrealistic assumption because the defensive capability and anti-predatory communication ability is viewed as a closely interacting pair in some systems (Sword et al., 2000; Sherratt, 2002; Broom, Speed & Ruxton, 2005; Speed, Ruxton & Broom, 2006).

However, allowing the evolution of repulsive taste in our model gives unnecessary complications with minimal benefit. Since the repulsive taste gives one-sided benefit in context of predator deterrence, simply allowing this component to vary is not a proper way of modeling its evolution. It must be accompanied by properly simulated mechanisms of defense cost, which need to bring in an array of assumptions about prey physiology and anatomy, as seen in the theories and discussions introduced above. Furthermore, prey defense can evolve without help of aposematism; chemical, mechanical, or behavioral qualities for antipredator defense could be beneficial in many other contexts as well. This complexity of the real world adds to the difficulty of expanding our model by including defense evolution and the associated mechanisms of cost-benefit tradeoffs of defense. Therefore, in our model we decided to set prey defense to a constant value set by the user (repulsive taste value). We hope that the achieved simplicity and universality of the model may be viewed as a strength for asking specific questions that focus on evolution of various signaling strategies.

We also viewed another omitted variable, the common maintenance cost of both the permanent and switchable signals, as a similar unnecessary complication. Unlike the signal ‘switchability’ maintenance that needs to be balanced against the dynamic benefit of the switchability, the static signal maintenance is a common penalty for both switchable and permanent aposematism. This had lower priority within our primary purpose: our question was focused on the evolution of the switchable aposematic signals, not the question of aposematism versus non-aposematism. For readers who are interested in the detailed mechanisms governing the latter type of evolutionary balance, we would like to recommend other existing theories over ours; Mappes, Marples & Endler (2005) and Ruxton et al. (2018) could serve as good entry points.

Finally, for the purpose of this particular paper, we deliberately configured the software to exclude any startling (“deimatic”) or learning facilitation effects of the switched signals. In the setups used in this paper, the strength of the signal being switched on is the only relevant factor in predator psychology; the fact that it was switched does not have any effect except that it imposes some cost to the prey. This decision helped us to study the cost-saving aspects of switchable aposematism without further complications. However, this assumption is far from reality (Umbers, Lehtonen & Mappes, 2015; Kang et al., 2016; Ruxton et al., 2018), and the model predicts that the switchable aposematism would be more favored if startle and facilitation effects could provide survival advantages (Fig. S1).

## Conclusions

In summary, our simulation provided support for many hypotheses about the diversity of aposematic strategies, especially focused on signal switchability. These include, but are not limited to, the following insights. First, the evolution of pre-attack switchable aposematism may require moderate range of predator learning speed, high basal detectability of the prey, and moderate to high level of signaling penalty. Second, the post-attack switchable aposematism may be favored under relatively low level of predator learning, low prey basal detectability, high signaling penalty. Third, high predator turnover combined with fast learning speed, a condition which produces a mix of naïve and experienced predators, may facilitate evolution of post-attack aposematism. Fourth, higher cost related to signal switching may lead to the disappearance of post-attack aposematism while forcing pre-attack aposematism to be fixed and permanent. During our study, we developed an individual-based modeling framework that can be used to explore a variety of questions regarding aposematism. This product is capable of simulating a number of variables that are not covered in this article, and we hope that it will inspire scientists and educators to further study the evolutionary biology of aposematic signals.

## Supplemental Information

10.7717/peerj.8915/supp-1 Supplemental Information 1 *ApoSim* model file (NetLogo 5.3.1 required) including the program code.

10.7717/peerj.8915/supp-2 Supplemental Information 2 Raw data for Figure 3 and Supplementary Figure 1: the most abundant strategy in each model run.

10.7717/peerj.8915/supp-3 Supplemental Information 3 Supplemental Figures and the *ApoSim* user manual.

## Additional Information and Declarations

### Data Availability

The following information was supplied regarding data availability: The source code and graphical interface and the raw data from the model runs are available in the Supplemental Files.
