# Supplementary material for: Evolution of switchable aposematism: insights from individual-based simulations
Source: PeerJ. 2020 Apr 10;8:e8915. doi: 10.7717/peerj.8915 (PMC7153555; doi:10.7717/peerj.8915)
Supplement: Supplemental Information 3 [file peerj-08-8915-s003.docx]

**
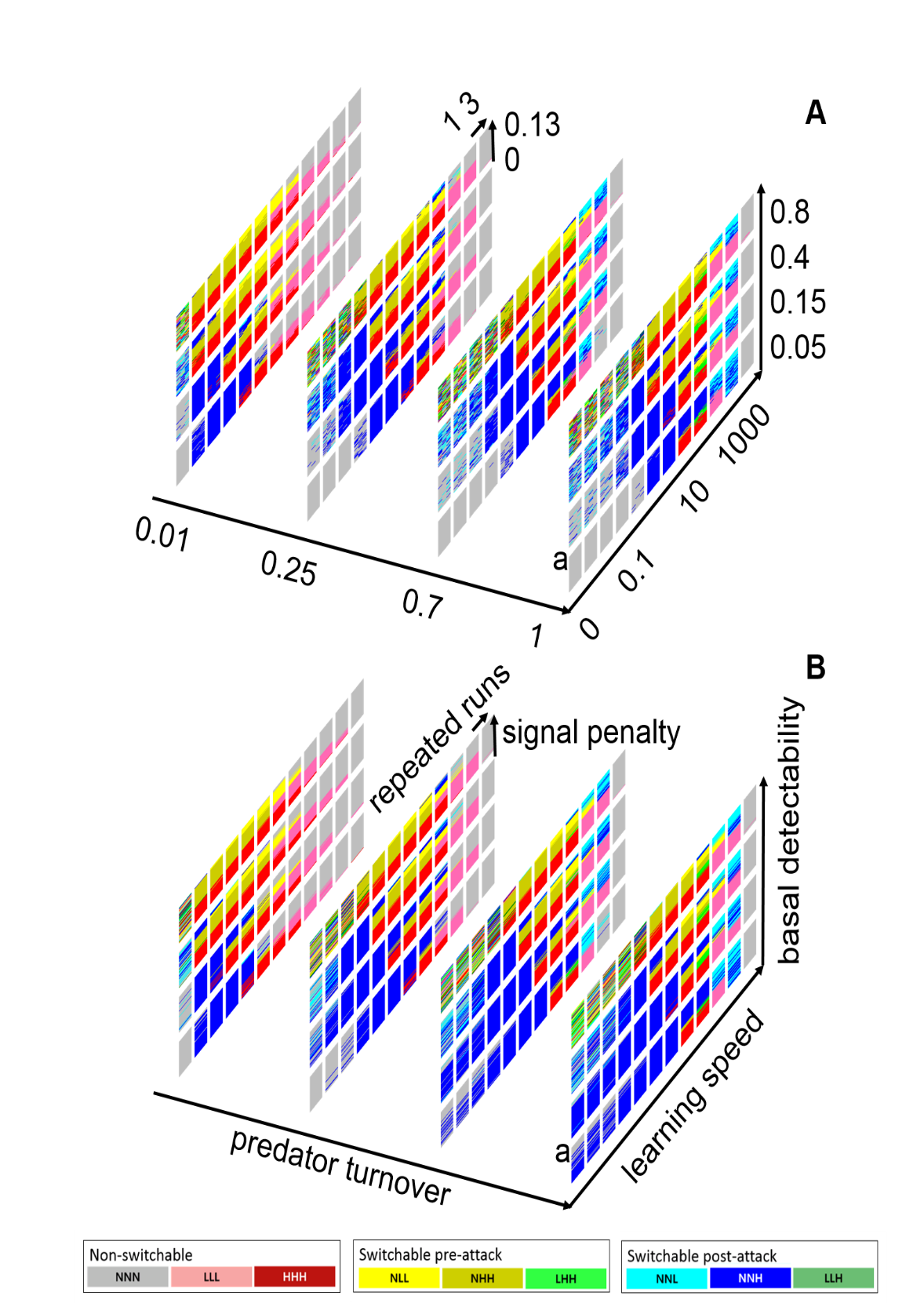
**

**Supplementary Figure 1.** An example of the model result meta-plots with startle and learning facilitation effects enabled. (A) Results of simulations with STARTLE-EFFECT 0.2, HABITUATION-SPEED 0.2, LEARNING-AID-EFFECT 0.2, along with all the other variables identical to the main text **Figure 3B**. (B) Reproduced main text **Figure 3B** for easier comparison. (a) An example of a condition where the startle and the learning-aid effect change the simulation outcome. Post-attack strong signals were more favored when startle and learning-aid effects were present. The difference was most noticeable in high **predator turnover**, low **basal detectability**, and low **learning speed**. This figure is given as an example of how the program *ApoSim* can be used to simulate effects that are not covered in the main text.

***
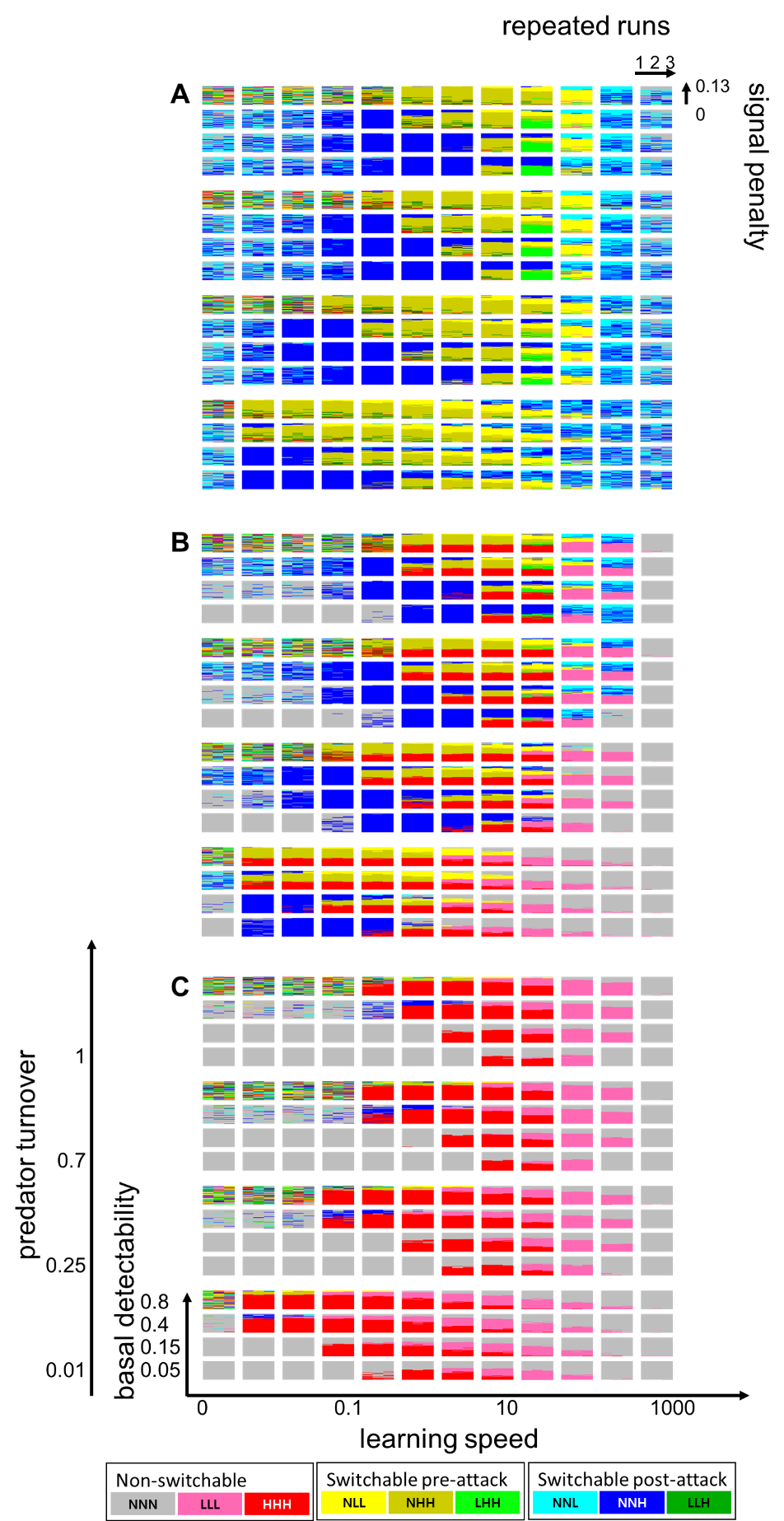
***

**Supplementary Figure 2.** The model result meta-plots represented in 2-dimensional view.

The contents of this figure is identical to the main text **Figure 3**; only the perspective is changed to provide an alternative visualization. Horizontal and vertical axes are the same in all three parts (A, B, C). A, the **switching cost** and the **switchability maintenance cost** are both none; B, there are moderate costs for both; C, the costs are prohibitively high. For more detail about the three levels of penalty of switchable signals, see the main text **Table 2**.


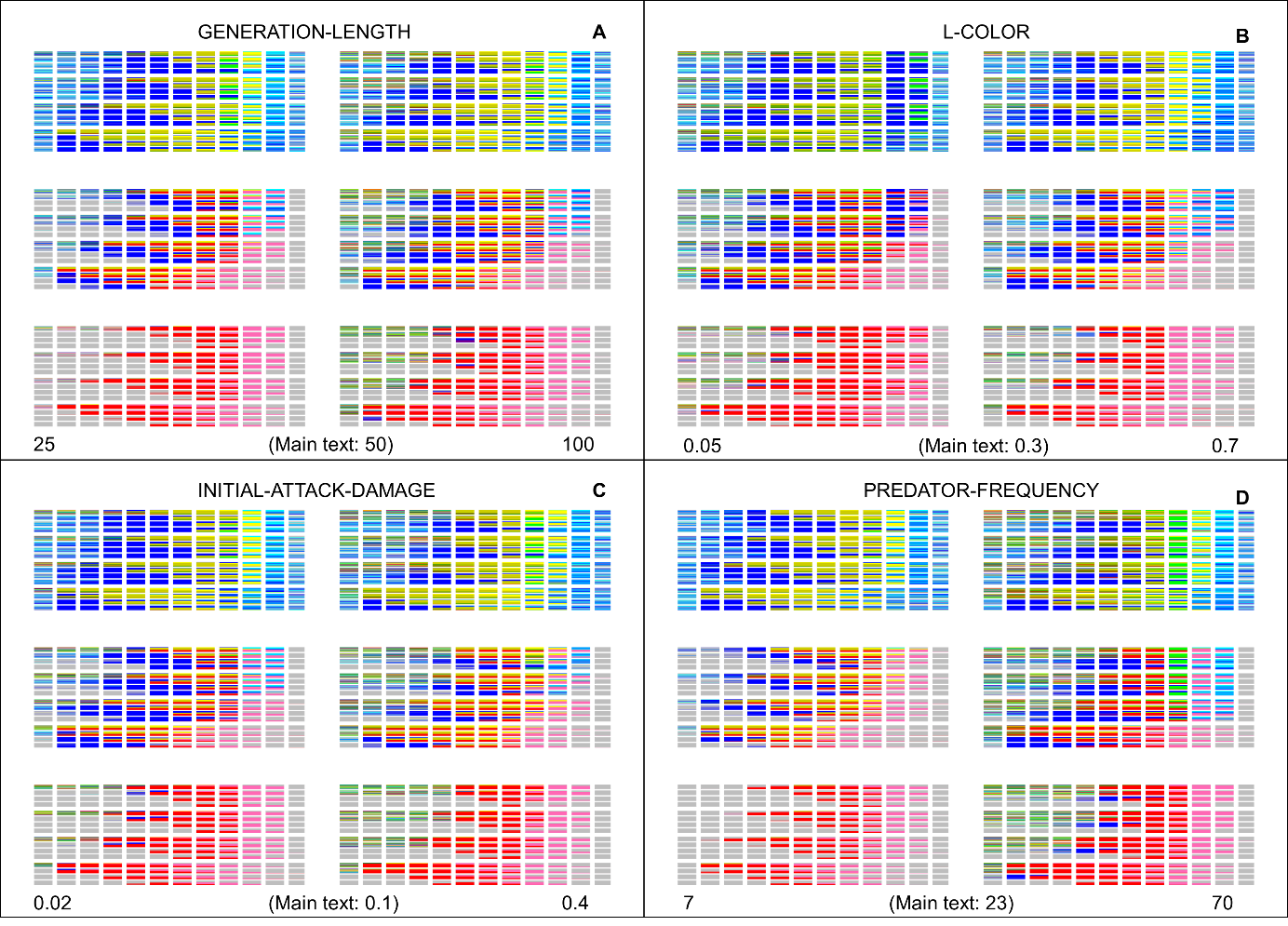


**Supplementary Figure 3.** Result of parameter-space bracketing.

(A) Generation length varied. (B) Low-signal (“L-COLOR”) varied. (C) Initial attack damage varied. (D) Predator frequency varied. The simulation runs depicted in this figure are performed to demonstrate the general robustness of the main text result. Below each subpanel, variable levels used for both extreme cases are given at the leftmost and rightmost corners, in addition to the level used in the main text in the middle. These four variables were chosen for this analysis because they were expected to differentially impact the different signal strategies, and at the same time, because they were reasonably expected to vary considerably without altering the core nature of the aposematism-driven system. For the rest of the axes and color codes, refer to the main text **Figure 3** or the equivalent **Supplementary Figure 2**.

***ApoSim* User Manual (= Information Tab of the NetLogo model file)**

**## WHAT IS IT?**

This project models aposematism (anti-predatory warning signal), predator learning and memory, and prey evolutionary dynamics. This model's main strength lies in the modelling of switchable aposematic signals. Unlike permanently colored animals, some prey animals can manifest warning signals only when attacked. This ability to switch signals can provide unique advantages, because the highly detectable signal can be hidden when not necessary. The model’s approach involves setting up cognitive and ecological characteristics of predators and determining the prey’s evolutionarily stable (“winning”) strategy(-ies), from among a user-defined set of prey signaling strategies in the specific ecological conditions defined by several types of costs of signaling.

**## HOW IT WORKS**

The accompanying article (Song et al. submitted) explains the internal rules the prey / predator individuals follow in detail. The following paragraphs summarize the paper's Method section briefly, but it is strongly recommended to read the full paper instead of this information tab.

The prey individuals can have three states, resting, approached, or attacked. In the 'approached' state the prey can give pre-attack signal to the predator. In the 'attacked' state the prey can give post-attack signal. The prey signal intensity is expressed at three levels, none, low and high. Therefore, the prey behavioral strategy is modeled as a sequence of three input-output pairs like the following: [(resting: None), (approached: Low), (attacked: High)]. The above sequence can be further summarized as three-character notation, "NLH." This gives the user the opportunity to define a variety of possible behavioral strategies in the RULES-LIST window. For example NNN indicates non-signaling prey, and LLL or HHH indicates a typical fixed aposematism at a lower or higher level of signal intensity, and NNH indicates post-attack displays of high intensity, and NHH indicates pre-attack displays of high intensity, etc. The behavioral strategy must have 3 letters, and otherwise the program will give an error.

Predators have two attack opportunities, 'initial attack' and 'final attack.' The prey's pre-attack signal is given before the initial attack, and it can subsequently influence the probability of later attacks. Likewise, the post-attack signal is given after the initial attack and before the final attack. The post-attack signal can subsequently influence the probability of the final attack.

The basic time unit of the simulation is the 'interaction frame.' Each frame includes three 'time steps.' The Step 1 is when the prey shows a pre-attack signal if a predator is present in the same grid. The Step 2 is when the initial attack occurs and when the post-attack signal is given. The Step 3 is when the final attack occurs.

Predators have 'aversive memory' that increases after initial- or final attack events. If a predator has high level of aversive memory, it has lower probability of attacking the prey. The speed of memory buildup and the efficacy of retrieval are affected by a number of factors, described in Song et al (submitted). The predator's ‘decision’ to attack or not affects the 'survival chance' of the prey, which in turn determines the prey’s success of reproduction.

In the reproduction stage, prey individuals differentially reproduce based on their survival chance and the existing generation dies out. At the same time, the predator population is refreshed by the adjustable variable 'predator turnover.'

During the interaction, the prey may pay a variety of costs that lower its survival chance. First, there is a 'switching cost' that is paid every time the signal intensity changes. Second, there is a 'switchability maintenance cost' that is continuously being paid if the prey has the ability to change the signal intensity. Third, 'signal penalty' is a cost paid during the presentation of the signal. Finally, the 'initial attack damage' and the 'final attack damage' are costs that are applied when the predator performs respective actions.

From the prey's viewpoint, the main benefit of a stronger signal is that, combined with the adjustable variable ‘learning speed’, it amplifies the buildup and retrieval of the predators’ aversive memory.

The main drawback of a stronger signal is that it can betray the prey's position to predators. The 'basal detectability' variable governs how the prey detectability changes depending on the signal intensity.

The detailed algorithms of the outlined model mechanics are available in the paper (Song et al. submitted).

**## HOW TO USE IT**

The model interface has three buttons, 'Play/Pause,' 'One Frame Forward,' and 'Apply Parameters and Generate World.' For the first-time users, it is recommended to click the 'Apply Parameters and Generate World,' wait for the world generation and then click the 'Play/Pause' button. This will help the users to become familiar with the workings of the model under the default parameter settings. It would be necessary to accelerate the model speed to actually visualize the evolutionary process.

The most important reporting interface is the 'Rules Histogram' plot. Relative abundance of each behavioral strategy is displayed here, and the strategies are sorted by the order specified in the RULES-LIST. In the model view, prey individuals are represented by 'butterfly' shapes, and the predators are depicted as yellow 'default' shapes. Depending on the signal intensity (none, low or high), the color of the prey changes from black to dark red to bright red.

The rest of the interface elements are outlined below.

Number of Rules monitor displays the number of behavioral strategies specified via RULES-LIST editing interface.

Generation monitor displays the number of prey generations that have passed.

Predators monitor displays the number of predators present.

Preys monitor displays the number of prey present.

Frames monitor displays the number of interaction frames that have passed.

Average Aversion monitor displays the current average value of the aversive memory of predators.

Time Step monitor displays the current time step of the frame, 1, 2, or 3.

Predator Aversion histogram displays the current distribution of predator aversive memory.

The label displayed next to each prey individual in the world view represents the behavioral strategy of that prey, according to the order listed in the RULES-LIST.

PLOT-ON toggle button is used to suspend plot updating when computation speed is preferred over visualization.

As the model currently has 19 parameters that can be adjusted by the sliders, users are encouraged to test them one by one. The three 'World Parameters' need re-generation of the model world to take effect, but the other 16 should immediately affect the model.

**WORLD-SIZE**

The size of the world. Theoretically no limits. The larger the world the slower is the program running speed, but the outcomes will have better reproducibility.

**PREDATOR-FREQUENCY**

Number of predators divided by the number of all patches. This may be viewed as a degree of saturation of the habitat with the predators, or as a proxy for density of predators.

**PREY-FREQUENCY**

Number of prey individuals divided by the number of all patches. This may be viewed as a degree of saturation of the habitat with the prey, or as a proxy for density of prey. Their relative density can play an important role in aposemtism evolution, as the encounter rate can determine whether the learning can be achieved with an acceptable risk(Sword 1999)

**GENERATION-LENGTH**

Number of interaction frames per prey generation. As the effect of GENERATION-LENGTH is generally proxied by LEARNING-SPEED (whether the predators can completely learn during the lifetime), it is advisable to use this variable as a mean of computational optimization only (giving more time vs. a larger world)

**BASAL-COGNITIVE-CUE**

Signal-independent cue from prey to predators. It corresponds to prey phenotypic traits, except the signaling, that affect the predator learning and predator memory retrieval. If BASAL-COGNITIVE-CUE is 1, the predator learning and retrieval are not affected by signal intensity at all. If BASAL-COGNITIVE-CUE is 0, the predator learning and retrieval are entirely dependent on the signal (neither learning nor retrieval can occur if there is no signal). Predators are shown to learn to avoid non-conspicuous defended prey, albeit at the slower speed (Kang et al. 2016). Therefore, it is important to model diverse predator-prey systems with different ‘basal’ learning speed using this variable.

**L-COLOR**

Signal intensity between 0 and 1 at the 'low' level signal.

**H-COLOR**

Signal intensity between 0 and 1 at the 'high' level signal.

**BASAL-DETECTABILITY**

The chance of prey being discovered when no signal is present. If BASAL-DETECTABILITY is 1, the prey is always discoverable regardless of the signal. If BASAL-DETECTABILITY is 0, the prey is invisible when no signal is given. This variable is analyzed in detail in the accompanying article (Song et al. submitted). Users might be interested in other discussions related to the detectability of non-signaling prey (Broom et al. 2010; Umbers and Mappes 2015; Willink et al. 2013).

**BAD-TASTE**

The strength of the aversive unconditional stimulus felt by the predators. Currently this single parameter controls two separate mechanics. First, it determines the learning-independent motivation drop after the initial attack. When BAD-TASTE is 0, the predator motivation would be unaffected unless some other effects are present. If BAD-TASTE is 1, the predator motivation would drop to zero after initial attack regardless of any other effects.

Second, it affects the aversive memory acquisition. If BAD-TASTE is 0, the predator would not be able to learn any aversion. If BAD-TASTE is 1, the predator learning is faster (how fast it is would be determined by a number of other factors).

**PREDATOR-TURNOVER**

The portion of predator population that are replaced with naive individuals per each prey generation. This variable is analyzed in detail in the accompanying article (Song et al. submitted). Predator turnover is one of the most prominent mechanisms that can result in a mix of naïve and experienced/educated predators (Endler and Mappes 2004), which may lead to diverse effects in the evolution of aposematism.

**LEARNING-SPEED**

The main coefficient that largely determines the speed of aversive memory buildup. If LEARNING-SPEED is 0.0, no aversion learning can occur. If LEARNING-SPEED is 1.0, then a single experience with a prey of BAD-TASTE 1.0 and signal intensity 1.0 will lead to 1.0 increase in aversive memory. LEARNING-SPEED does not have an upper bound, and an arbitrarily high value of LEARNING-SPEED will enable arbitrarily fast aversive memory buildup. For the detail of the processes regarding aversive memory and its implication in the evolution of switchable aposematism, reading the accompanying article (Song et al. 2019) is recommended. There are also pertinent empirical (Kang et al. 2016) and theoretical (Puurtinen and Kaitala 2006; Speed 2001) discussions of this variable.

**SIGNAL-PENALTY**

The cost (drop in survival chance) unconditionally paid by the prey due to the signal intensity. This variable summarizes all types of signal cost except the detectability cost. If SIGNAL-PENALTY is 0.0, there is no inherent cost in high-intensity signal except that it can draw predators' attention. If SIGNAL-PENALTY is 1.0, displaying an intensity=1.0 signal will instantly drop its survival chance to zero. The accompanying article (Song et al. submitted) discusses this variable in more detail. The costs of signal other than detectability can have a variety of effects (Blount et al. 2009) on the evolution of aposematism, including interactions outside of this model’s scope.

**SWITCHING-COST**

The cost (drop in survival chance) paid by the prey whenever its signal intensity changes. If SWITCHING-COST is 0.0, there is no cost in signal switching. If SWITCHING-COST is 1, altering from intensity=0.0 to intensity=1.0 or vice versa will instantly drop its survival chance to zero. Along with the SWITCHABILITY-MAINTENANCE-COST below, this variable has been explored in the accompanying article (Song et al. submitted). A previous theory (Broom et al. 2010), while not directly related to switchable signals, provide a good perspective about the potential of such cost affecting aposematism evolution.

**SWITCHABILITY-MAINTENANCE-COST**

The cost (drop in survival chance) unconditionally paid by the prey if the prey has a potential to change signal intensity. If SWITCHABILITY-MAINTENANCE-COST is 0.0, prey that does not actually switch signals would pay no cost even if they have the ability to do so. If SWITCHABILITY-MAINTENANCE-COST is 1.0, prey that are capable of switching signals will suffer instant elimination of any survival chance, regardless whether such switching behavior actually occurred or not. Along with the SWITCHING-COST above, this variable has been explored in the accompanying article (Song et al. submitted). A previous theory (Broom et al. 2010), while not directly related to switchable signals, provide a good perspective about the potential of such costs to affect evolution of aposematism.

**INITIAL-ATTACK-DAMAGE**

The cost (drop in survival chance) paid by the prey each time a predator conducts 'initial attack (the attack before seeing post-attack signal)' on it. If INITIAL-ATTACK-DAMAGE is 0.0, there is no damage from the attack. If INITIAL-ATTACK-DAMAGE is 1.0, a single attack event eliminates all survival chance. The survival rate after being attacked has long been regarded as a key factor in aposematism evolution (Wiklund and Jarvi 1982). The difference of this variable from the FINAL-ATTACK-DAMAGE below can arise from predator response to mimicry (Gamberale-Stille and Guilford 2004).

**FINAL-ATTACK-DAMAGE**

The cost (drop in survival chance) paid by the prey each time a predator conducts 'final attack (the attack after seeing post-attack signal)' on it. If FINAL-ATTACK-DAMAGE is 0.0, there is no damage from the attack. If FINAL-ATTACK-DAMAGE is 1.0, a single attack event eliminates all survival chance. The survival rate after being attacked has long been regarded as a key factor in aposematism evolution (Wiklund and Jarvi 1982). The difference of this variable from the INITIAL-ATTACK-DAMAGE above can arise from predator response to mimicry (Gamberale-Stille and Guilford 2004).

**LEARNING-AID-EFFECT**

Facilitation of aversive memory buildup due to change in signal intensity. If LEARNING-AID-EFFECT is 0.0, switched signals and permanent signals with the same intensity have same effect on predator learning. If LEARNING-AID-EFFECT is 1.0, a switched signal that had undergone intensity jump of 1.0 would have twice as great an effect as the equivalent permanent signal. This value has no upper bound, and infinitely high value of LEARNING-AID-EFFECT will have infinite advantage to switched signals compared to permanent signals. The effect of learning facilitation by switchable aposematism has been empirically tested (Kang et al. 2016).

**STARTLE-EFFECT**

Drop of prey attractiveness (chance to decide to attack) due to switching of the signal. If STARTLE-EFFECT is 0.0, signal switching itself does not decrease the attack frequency. If STARTLE-EFFECT is 1.0, prey that has changed the signal intensity by 1.0 would lose all attractiveness to predators that have seen such change for the first time. Note that the effect of startle can be attenuated by habituation as specified by the HABITUATION-SPEED variable below. The startle display (deimatism) and the warning signal (aposematism) often share critical components or are known to simultaneously govern predator psychology (Bates and Fenton 1990; Schlenoff 1985).

**HABITUATION-SPEED**

Speed of loss of effective STARTLE-EFFECT. The effective STARTLE-EFFECT approaches zero as the predator accumulates the observations of prey signal switching. If HABITUATION-SPEED is 0.0, the effective STARTLE-EFFECT never changes from the value specified with the interface slider. If HABITUATION-SPEED is 1.0, a single experience of jump of signal intensity by 1.0 will remove all effective STARTLE-EFFECT for the rest of the predator's lifetime. Different systems have been shown to have different habituation characteristics (Bates and Fenton 1990; Olofsson et al. 2012)

**## WHAT TO DO WITH IT**

This model is built in NetLogo 5.3.1, which features a systematic parameter-sweeping tool called BehaviorSpace (<https://ccl.northwestern.edu/netlogo/docs/behaviorspace.html>). In order to explore a wide range of model parameters, the user may need to set up an ‘experiment’ as per the procedures outlined in the web manual. The accompanying article (Song et al. submitted) is also centered on four BehaviorSpace experiments, which created the Figure 2A, 2B, 2C and the Supplementary Figure 1 of the paper, respectively. The user can use these BehaviorSpace experiments (via Tools->BehaviorSpace) as starting examples to design their own ones.

**## CREDITS AND REFERENCES**

Bates DL, Fenton MB. 1990. Aposematism or Startle - Predators Learn Their Responses to the Defenses of Prey. Canadian Journal of Zoology-Revue Canadienne De Zoologie. 68(1): 49-52.

Blount JD, Speed MP, Ruxton GD, Stephens PA. 2009. Warning displays may function as honest signals of toxicity. P Roy Soc B-Biol Sci. 276(1658): 871-877.

Broom M, Higginson AD, Ruxton GD. 2010. Optimal investment across different aspects of anti-predator defences. J Theor Biol. 263(4): 579-586.

Endler JA, Mappes J. 2004. Predator mixes and the conspicuousness of aposematic signals. Am Nat. 163(4): 532-547.

Gamberale-Stille G, Guilford T. 2004. Automimicry destabilizes aposematism: predator sample-and-reject behaviour may provide a solution. P Roy Soc B-Biol Sci. 271(1557): 2621-2625.

Kang C, Cho HJ, Lee SI, Jablonski PG. 2016. Post-attack Aposematic Display in Prey Facilitates Predator Avoidance Learning. Frontiers in Ecology and Evolution. 4.

Olofsson M, Eriksson S, Jakobsson S, Wiklund C. 2012. Deimatic Display in the European Swallowtail Butterfly as a Secondary Defence against Attacks from Great Tits. Plos One. 7(10).

Puurtinen M, Kaitala V. 2006. Conditions for the spread of conspicuous warning signals: A numerical model with novel insights. Evolution. 60(11): 2246-2256.

Schlenoff DH. 1985. The Startle Responses of Blue Jays to Catocala (Lepidoptera, Noctuidae) Prey Models. Anim Behav. 33(Nov): 1057-1067.

Speed MP. 2001. Can receiver psychology explain the evolution of aposematism? Anim Behav. 61: 205-216.

Sword GA. 1999. Density-dependent warning coloration. Nature. 397(6716): 217-217.

Umbers KDL, Mappes J. 2015. Postattack deimatic display in the mountain katydid, Acripeza reticulata. Anim Behav. 100: 68-73.

Wiklund C, Jarvi T. 1982. Survival of Distasteful Insects after Being Attacked by Naive Birds - a Reappraisal of the Theory of Aposematic Coloration Evolving through Individual Selection. Evolution. 36(5): 998-1002.

Willink B, Brenes-Mora E, Bolanos F, Prohl H. 2013. Not Everything Is Black and White: Color and Behavioral Variation Reveal a Continuum between Cryptic and Aposematic Strategies in a Polymorphic Poison Frog. Evolution. 67(10): 2783-2794.
